# Angiopoietin-like 4 (ANGPTL4) Protein and Its Relation to Lipids Challenges the Prognostic Performance of B-Type Natriuretic Peptide (BNP) Transcontinentally in Patients with Suspected Coronary Chest Pain, Suggesting Their Combined Use in Risk Prediction

**DOI:** 10.3390/ijms27219966

**Published:** 2026-11-06

**Authors:** Dennis W. T. Nilsen, Ricardo A. Leon de la Fuente, Reidun Aarsetoey, Patrycja A. Naesgaard, Patricio F. Gallo, Silvia Dib Ashur, Trygve Brugger-Andersen, Volker Poenitz, Heidi Grundt, Annika Elisabet Michelsen, Pål Aukrust, Harry Staines, Thor Ueland

**Affiliations:** 1 Department of Cardiology, Stavanger University Hospital, 4068 Stavanger, Norway; ricaleon@gmail.com (R.A.L.d.l.F.); patrycja.anna.nesgaard@sus.no (P.A.N.); hagerman@online.no (T.B.-A.); volker.poenitz@lyse.net (V.P.); 2 Department of Clinical Science, University of Bergen, 5021 Bergen, Norway; magnea.heidi.jonsdottir.grundt@sus.no; 3 Internal Medicine Program, Hospital Papa Francisco, Salta A4400, Argentina; 4 Department of Cardiology, Cardiovascular Center of Salta, Salta A4406, Argentina; pfgallo@hotmail.com (P.F.G.); silviadibashur@gmail.com (S.D.A.); 5 Department of Respiratory Medicine, Stavanger University Hospital, 4068 Stavanger, Norway; 6 Faculty of Medicine, University of Oslo, 0318 Oslo, Norway; a.e.michelsen@medisin.uio.no (A.E.M.); paukrust@ous-hf.no (P.A.); thor.ueland@medisin.uio.no (T.U.); 7 Research Institute of Internal Medicine, Oslo University Hospital, Rikshospitalet, 0372 Oslo, Norway; 8 Section of Clinical Immunology and Infectious Diseases, Oslo University Hospital, Rikshospitalet, 0372 Oslo, Norway; 9 Sigma Statistical Services, Balmullo KY160BD, UK; harry.j.staines@gmail.com

**Keywords:** angiopoietin-like 4 (ANGPTL4) protein, B-type natriuretic peptide (BNP), lipids including triglycerides (TG), acute coronary syndrome (ACS), all-cause mortality

## Abstract

BNP is predominantly synthesized in the ventricular myocardium in response to ventricular distension. ANGPTL4 modulates lipid metabolism, vascular integrity and angiogenesis. Both biomarkers serve as prognostic indicators in acute coronary syndrome (ACS). The purpose of this study is to compare the prognostic weighting of ANGPTL4 with BNP in a cross-continental setting and to assess the former’s association with circulating lipids in patients with suspected coronary chest pain. Patients were recruited in Norway (NOR) and Argentina (ARG) and matched exactly by propensity scoring according to age, sex and previous use of statins, yielding 507 subjects in each group. Cox regression models were fitted for standardized log_e_ ANGPTL4 and log_e_ BNP, respectively, with all-cause mortality within 5 years as the dependent variable. Receiver Operating Characteristic (ROC) analysis was applied for the area under curve (AUC). Death rates were similar: (ARG = 20.1%) and (NOR = 22.7%), *p* = 0.320. ANGPTL4 and BNP were independently associated with all-cause mortality in each cohort. Concentrations of ANGPTL4 and triglycerides (TG) were higher, and total cholesterol (TC), LDL-C and HDL-C were lower in ARG compared to NOR (each *p* < 0.001). ANGPTL4 correlated negatively with each lipid fraction and positively with C-reactive protein (CRP) but did not correlate with body mass index (BMI) in the cohorts combined. AUC for ANGPTL4 outperformed BNP in ARG, and the reverse was observed in NOR. However, AUC for ANGPTL4 in the combined cohorts was 0.718 (95%CI 0.680–0.756) and for BNP it was 0.730 (95%CI 0.691–0.769). Their different pathogenic pathways may improve prognostic information, with a pattern that may differ between continents.

## 1. Introduction

The angiopoietin-like protein 4 (ANGPTL4) is a secreted protein with structural similarity to angiopoietins [1]. ANGPTL4 converts lipoprotein lipase (LPL) to inactive monomers [2,3] and inhibits the breakdown on luminal capillary surfaces of triglycerides (TGs) [4], resulting in reduced uptake of TG-derived fatty acids (FAs) into adipose and other tissues, such as the myocardium and skeletal muscle [5]. TG-derived FAs bind to the N-terminal end of ANGPTL4, effectively reversing LPL inhibition and enabling FA uptake, followed by an upregulation of ANGPTL4 [6,7]. In addition to its regulation of TG degradation, ANGPTL4 impacts glucose and energy metabolism [8]. Through its C-terminal end it is involved in the regulation of angiogenesis and inflammatory responses [1,9].

Gain-of-function genetic variants in LPL and loss-of-function variants in its intravascular inhibitors, which include ANGPTL4, are associated with lower TG levels and lower coronary disease risk [10,11]. A genetic loss-of-function variant of ANGPTL4 (E40K) that is found in 3% of European Americans is associated with reduced plasma TG levels and increased high-density lipoprotein cholesterol (HDL-C) [12], but was—somewhat surprisingly—associated with increased coronary heart disease (CHD) risk [13]. The clinical implications of these findings are, however, unclear, and so far, no strong positive correlation has been found between plasma ANGPTL4 and plasma TG levels in human subjects [14,15]. Plasma ANGPTL4, however, was found to be significantly higher in subjects with the metabolic syndrome and predicted future cardiovascular events univariately and after adjustment for standard cardiovascular risk factors [16].

We have previously reported a significant association between ANGPTL4 and all-cause mortality in subjects admitted with chest pain of suspected coronary origin [17]. We found an independent association between ANGPTL4 and all-cause mortality in an Argentinian population, whereas only a trend was observed in a Norwegian population. In that study, the outcome data related to ANGPTL4 were adjusted for B-type natriuretic peptide (BNP), an established risk marker in patients with acute coronary syndrome [18,19]. However, we did not perform any direct comparison between these two markers in relation to outcome. This could be of particular interest, as differences in prognostic utility might be influenced by metabolic disturbances [20], a factor of particular relevance in an Argentinian population [21] and, as described above, in relation to ANGPTL4.

As metabolic differences exist between populations, our main objectives of this explorative clinical study were as follows: (1) To perform a prognostic weighting of ANGPTL4 as compared to that of BNP in relation to all-cause mortality in patients from two separate populations on different continents presenting with chest pain of suspected coronary origin and matched by propensity scoring according to age, sex and previous use of statins. (2) To investigate the relation of ANGPTL4 to circulating lipid fractions and other metabolic traits, such as diabetes type 2 and BMI. Furthermore, as these metabolic changes also may influence inflammation, we also related ANGPTL4 and BNP to hsCRP, assessed separately for the Argentinian and the Norwegian cohorts, respectively, and for the combined population.

Study participation was based on the same protocol in both countries. To further improve similarity, and taking into account cholesterol reducing therapy, patients in Argentina and Norway, respectively, were matched by propensity scoring according to age, sex and the previous use of statins, acknowledging residual intercontinental discrepancies which were adjusted for in multivariable analysis.

## 2. Results

### 2.1. Clinical Characteristics

ANGPTL4 levels were collectively available in 994 patients (ARG = 506, NOR = 488), and the corresponding number for BNP was 983 (ARG = 507, NOR = 476).

Baseline characteristics for the two cohorts are shown in Table 1. Age, sex and previous statin use were similar for the two groups. Median values of ANGPTL4 were significantly higher in the ARG as compared to the NOR population (3.74 vs. 2.89 ng/mL, *p* < 0.001), whereas the median BNP level was normal (<100 pg/ml) in both populations (86.7 vs. 64.0 pg/mL, respectively, *p* = 0.055). Box plots demonstrating between-country comparisons of ANGPTL4 and BNP are shown in Supplementary Figure S1a,b. TGs were significantly higher among the Argentinians (1.60 vs. 1.30 mmol/L, *p* < 0.001), whereas the other lipid fractions were lower [TC 4.60 vs. 5.50 mmol/L, HDL-C 1.00 vs. 1.30 mmol/L, LDL-C 2.67 vs. 3.46 mmol/L (*p* < 0.001 for all)]. The differences in lipid fractions are demonstrated by box plots in Supplementary Figure S2a–d.

The association between ANGPTL4 and BNP was stronger for Norwegian than Argentinian patients (Supplementary Figure S3a–c; Argentina: rs = 0.315, *p* < 0.001. Norway: rs = 0.438, *p* < 0.001), whereas the reverse was true for the association between ANGPTL4 and TnT (Supplementary Figure S4a–c; Argentina: rs = 0.299, *p* < 0.001. Norway: rs = 0.133, *p* = 0.003).

Comparing covariables between the two countries, several significant differences were noted; see Table 1 for *p*-values. Proportionally, there were significantly more patients with a history of MI and established CHD in the Norwegian as compared to the Argentinian cohort, whereas the opposite was noted for diabetes type 2, hypertension and smoking. The proportion of patients with established heart failure was similar, as was the rate of previous CABG, whereas the rate of previous PCI was 2.4 times higher in the Norwegian population. HsCRP was slightly higher and eGFR was lower in the Norwegians, and the Argentinians had a higher BMI. Furthermore, a higher proportion of Argentinians were being treated with angiotensin-converting-enzyme (ACE) inhibitors/angiotensin receptor blockers (ARBs), beta-blockers and acetylsalicylic acid (ASA).

The baseline quartile distributions of covariables according to quartiles of ANGPTL4 in the Argentinian and Norwegian populations are shown in Supplementary Table S1 and Supplementary Table S2, respectively.

### 2.2. Prognostic Utility of ANGPTL4 and BNP at 5 Years’ Follow-Up in the Argentinian Population

There was no significant difference in all-cause mortality at 5-year follow-up in the Argentinian (20.1%) compared to the Norwegian (22.7%) cohort of patients, *p* = 0.320. Cardiac death rates at 2 years were 7.80% for the Argentinian and 6.71% for the Norwegian population (*p* = 0.505), respectively.

**Figure 1:**
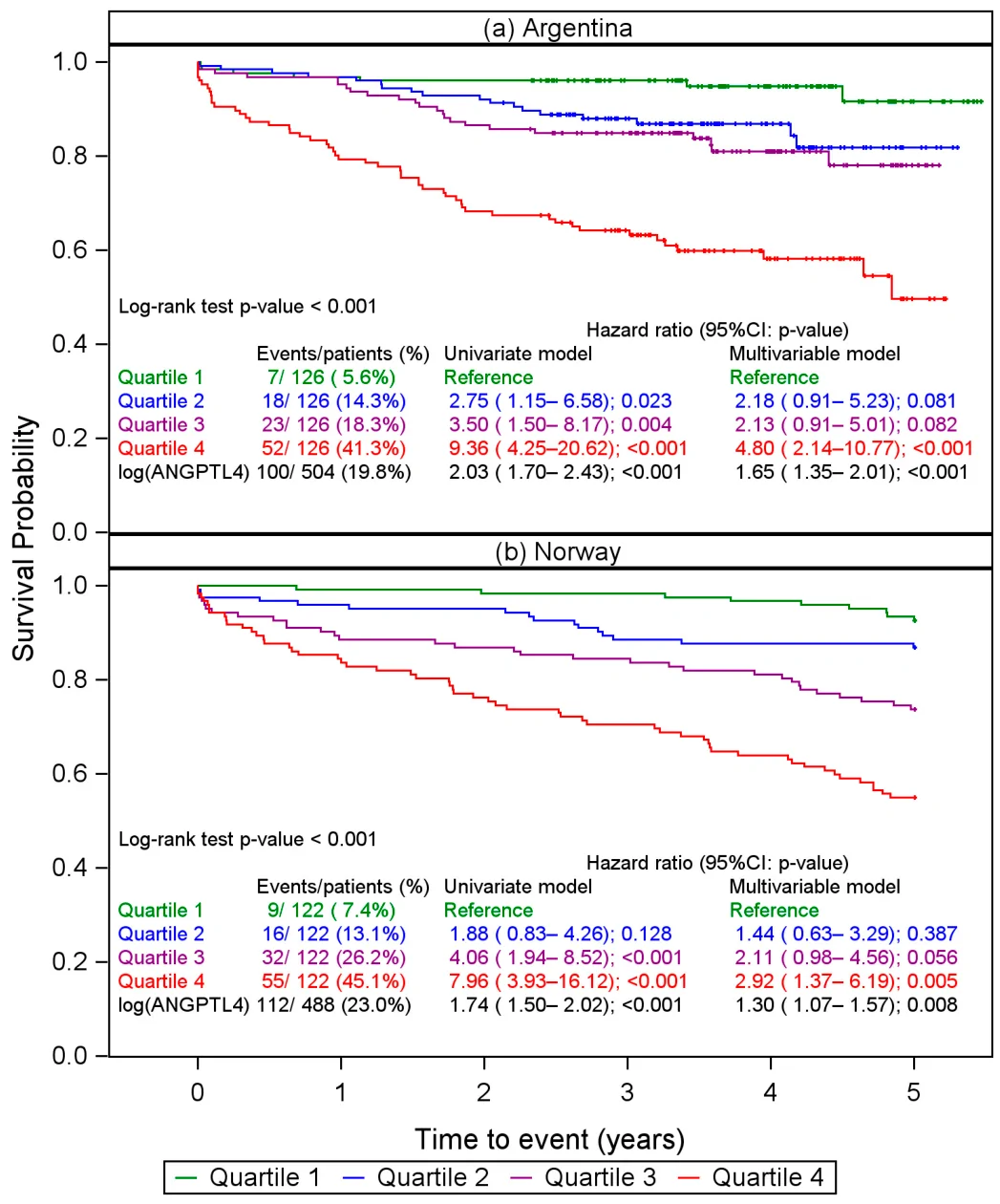
(**a**,**b**). Kaplan–Meier plots describing the prognostic utility of angiopoietin-like 4 (ANGPTL4) for 5 years’ all-cause mortality, arranged according to its quartiles in (**a**) 504 Argentinian and (**b**) 488 Norwegian patients admitted with acute chest pain of suspected coronary origin and matched by propensity scoring according to age, gender and previous statin use.

Applying continuous values in univariate analysis for assessment of the association between **ANGPTL4** and 5-year all-cause mortality in the Argentinian population yielded an HR of 2.03 (95%CI 1.70–2.43), *p* < 0.001. Comparing Q4 with Q1 in a quartile analysis resulted in an HR of 9.36 (95%CI 4.25–20.62), *p* < 0.001. The Kaplan–Meier curves displayed in Figure 1a were associated with a highly significant log-rank test (*p* < 0.001). After covariate adjustment, an HR of 4.80 (95%CI 2.14–10.77), *p* < 0.001 was obtained when comparing Q4 with Q1. Furthermore, a consistent independent association between ANGPTL4 and all-cause mortality was confirmed in the continuous multivariable adjusted analysis [HR 1.65 (95%CI 1.35–2.01), *p* < 0.001].

**Figure 2:**
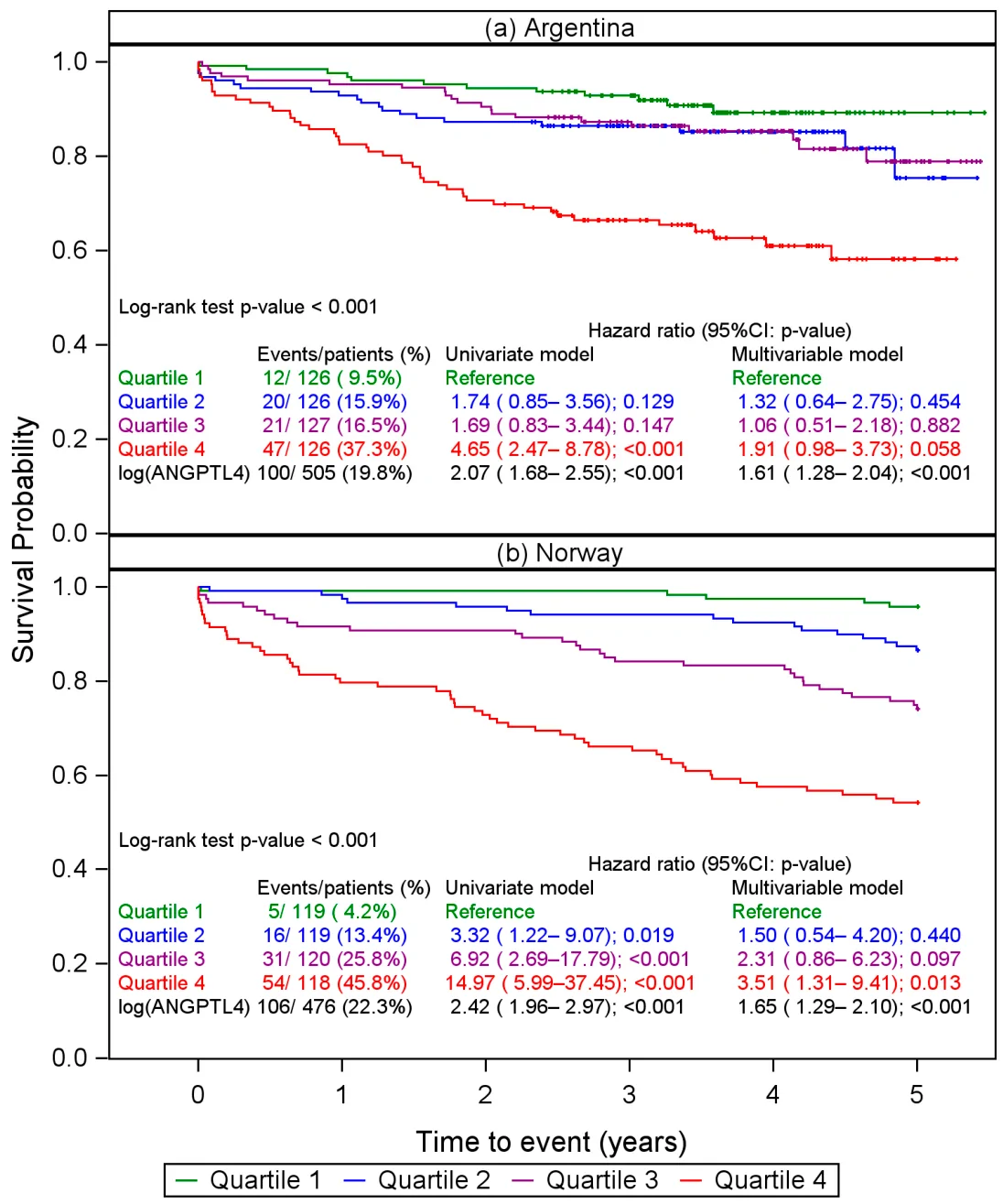
(**a**,**b**). Kaplan–Meier plots describing the prognostic utility of B-type natriuretic peptide (BNP) for 5-year all-cause mortality, arranged according to its quartiles in (**a**) 505 Argentinian and (**b**) 476 Norwegian patients admitted with acute chest pain of suspected coronary origin and matched by propensity scoring according to age, gender and previous statin use.

For **BNP** in the Argentinian cohort, univariate and multivariable analyses resulted in an HR of 2.07 (95%CI 1.68–2.55), *p* < 0.001, and HR 1.61 (95%CI 1.28–2.04), *p* < 0.001, respectively, using continuous values. The Kaplan–Meier curves displayed in Figure 2a were associated with a highly significant log-rank test (*p* < 0.0001). The results of the univariate and adjusted quartile analyses of the association between BNP and all-cause mortality are also depicted in Figure 2a.

### 2.3. Prognostic Utility of ANGPTL4 and BNP at 5 Years’ Follow-Up in the Norwegian Population

In univariate analysis, ANGPTL4 was also found to be associated with all-cause mortality in the Norwegian population. Applying continuous values, the univariate HR was 1.74 (95%CI 1.50–2.02), *p* < 0.001. In a quartile analysis, Q4 compared with Q1 resulted in an HR of 7.96 (95%CI 3.93–16.12), *p* < 0.001. The Kaplan–Meier curves displayed in Figure 1b were associated with a highly significant log-rank test (*p* < 0.001). After adjusting for covariates, an HR of 2.92 (95%CI 1.37–6.19), *p* = 0.005, was obtained when comparing Q4 with Q1. An independent association was consistently present between ANGPTL4 and all-cause mortality in the continuous multivariable adjusted analysis [HR 1.30 (95%CI 1.07–1.57), *p* = 0.008].

For **BNP** in the Norwegian cohort, applying continuous values in univariate and multivariable analyses, an HR of 2.42 (95%CI 1.96–2.97), *p* < 0.001, and an HR of 1.65 (95%CI 1.29–2.10), *p* < 0.001, respectively, was obtained. The Kaplan–Meier curves displayed in Figure 2b were associated with a highly significant log-rank test (*p* < 0.001). The results of the univariate and adjusted quartile analyses of the association between BNP and all-cause mortality are also depicted in Figure 2b.

### 2.4. Prognostic Utility of ANGPTL4 and BNP for All-Cause Mortality at 2 Years’ Follow-Up

Applying continuous values in univariate and multivariable analysis for the assessment of the association between **ANGPTL4** and 2-year all-cause mortality (14.3%) in the Argentinian population yielded an HR of 2.11 (95%CI 1.72–2.61), *p* < 0.001, and an HR of 1.71 (95%CI 1.34–2.18), *p* < 0.001, respectively. The corresponding results related to all-cause mortality (10.9%) in the Norwegian population were HR 1.83 (95%CI 1.48–2.26), *p* < 0.001, and HR 1.28 (95%CI 0.97–1.70), *p* = 0.086, respectively.

For the Argentinian patients, univariate and multivariable assessment of the association between **BNP** and all-cause mortality yielded an HR of 2.24 (95%CI 1.76–2.85), *p* < 0.001, and an HR 1.57 (95%CI 1.19–2.06), *p* < 0.001, respectively. The corresponding results in the Norwegian population were HR 2.69 (95%CI 1.97–3.65), *p* < 0.001, and HR 2.10 (95%CI 1.48–2.98), *p* < 0.001.

The Kaplan–Meier survival curves for ANGPTL4 and BNP in relation to 2-year all-cause mortality are shown in Supplementary Figures S5 and S6. An independent association between ANGPTL4 in Q4 compared to Q1 and all-cause mortality (*p* = 0.002) was demonstrated in the Argentinian cohort but did not reach statistical significance in the Norwegian cohort (*p* = 0.083), whereas the corresponding results for BNP were highly significant (*p* < 0.001), Similar results were obtained employing continuous values.

### 2.5. Weighting the Prognostic Utility of ANGPTL4 Compared with BNP at 5-Years’ Follow-Up

**Figure 3:**
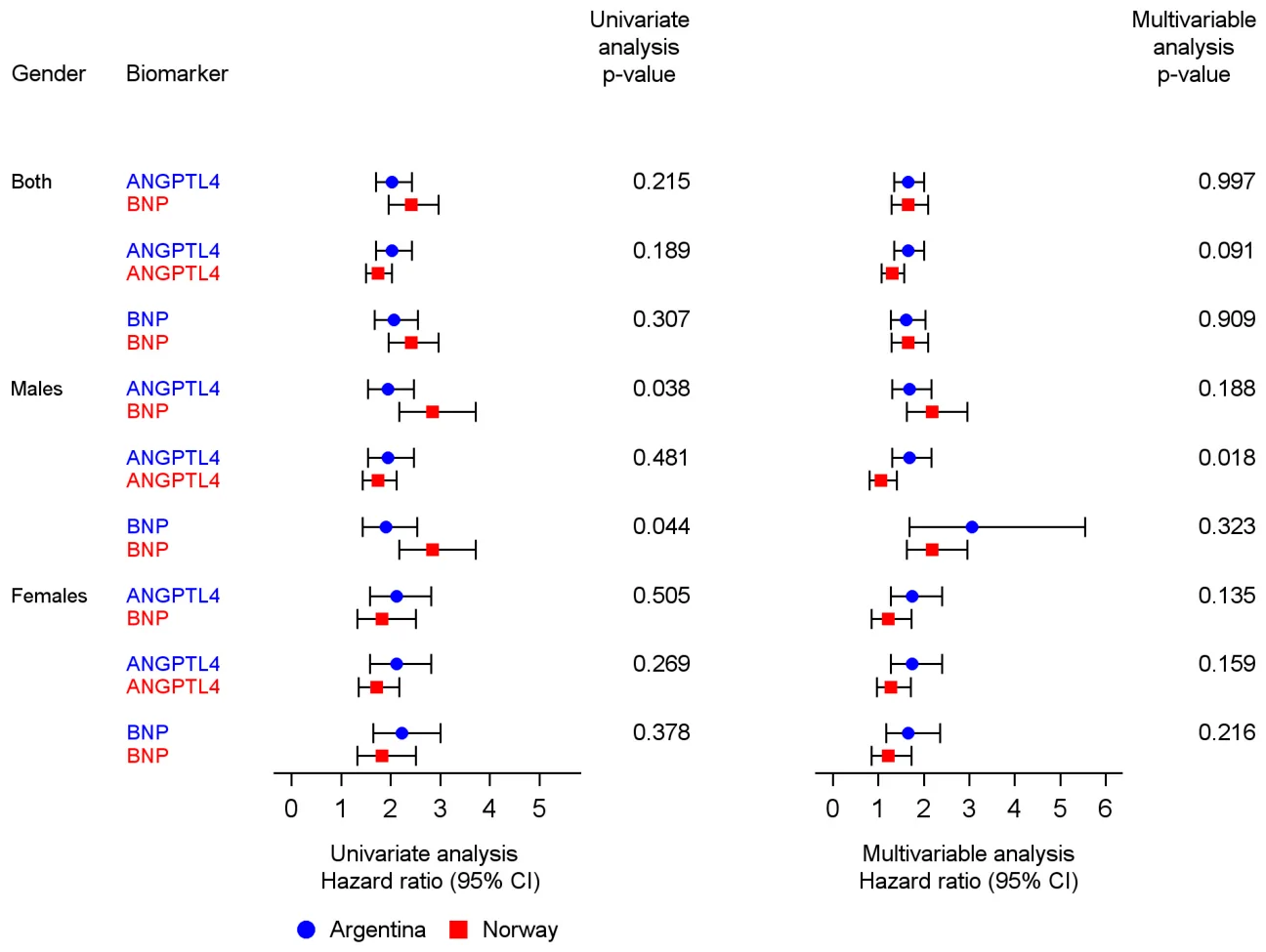
Forest plot comparisons of the prognostic utility of angiopoietin-like 4 (ANGPTL4) protein and brain natriuretic peptide (BNP), assessed country-wise and between-countries, for prediction of 5-year all-cause mortality in a cross-continental study including patients with acute chest pain of suspected coronary origin, recruited from Argentina and Norway, respectively, after matching the cohorts by propensity scoring according to age, sex and previous use of statins, yielding 507 subjects in each group. The figure contains comparative results for all, and for males and females, respectively. We did not adjust for multiple comparisons, and the results should be interpreted with caution. CI: confidence interval.

The results based on univariate and multivariable continuous values of inter-country comparisons of risk [HR (95%CI)] for 5-year all-cause mortality carried by the investigated biomarkers are shown in Figure 3. The overall univariate risk related to **BNP** levels appeared to be greater in the Norwegian compared with the Argentinian population, reaching statistical significance between the respective male populations (*p* = 0.044). However, the overall adjusted association between BNP and mortality was quite similar in both cohorts (*p* = 0.909) (Figure 3).

In the overall univariate analysis, BNP-associated risk of all-cause mortality in the Norwegian cohort tended to outweigh that of ANGPTL4 in the Argentinian cohort (*p* = 0.215). However, after adjustment, ANGPTL4-related risk in the Argentinian cohort [HR 1.65 (95%CI 1.35–2.01)] was similar to that of BNP in the Norwegian cohort [HR 1.65 (95%CI 1.29–2.10)], *p* = 0.997). Both were strongly and independently associated with 5-year all-cause mortality in their respective populations.

Comparison of the between-country adjusted risk related to continuous values of **ANGPTL4** for 5-year all-cause mortality, seemed to favour the use of ANGPTL4 in Argentina compared to Norway (*p* = 0.091), reaching statistical significance in men (*p* = 0.018).

**Figure 4:**
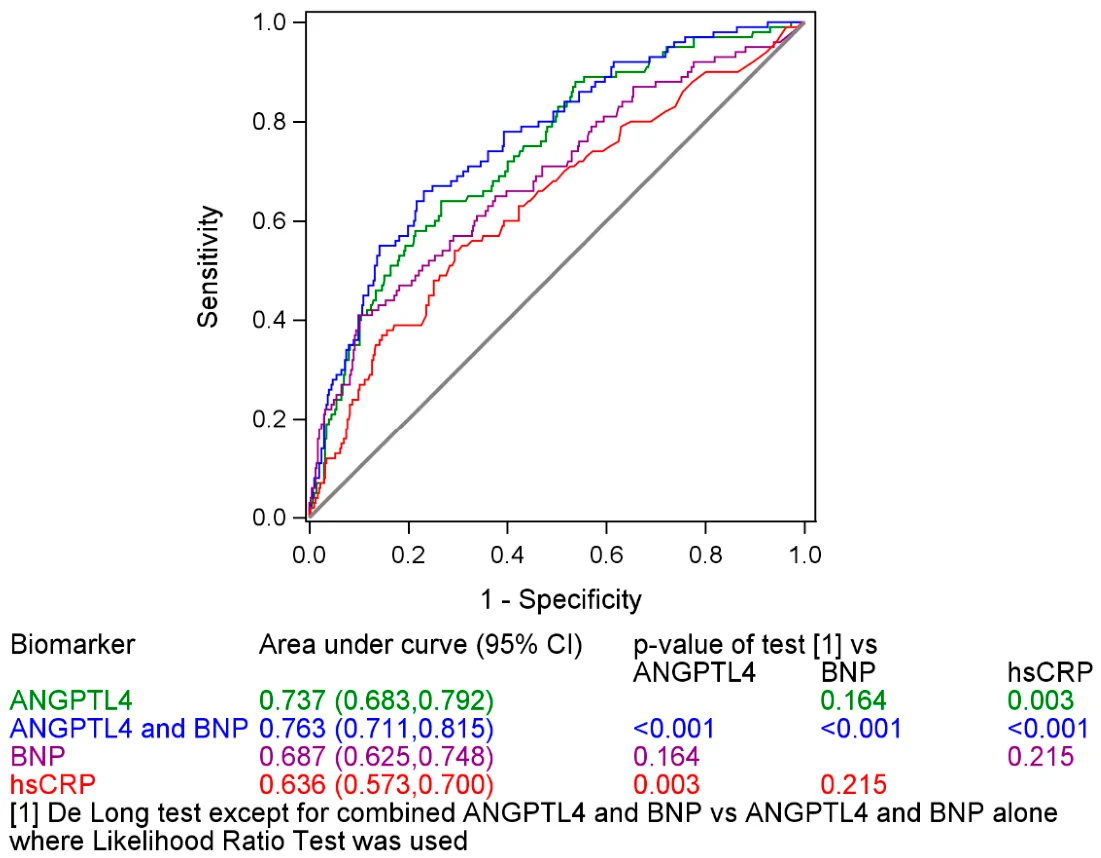
Receiver Operating Characteristic (ROC) curves and area under curve (AUC) (95% CI) values for ANGPTL4, BNP, ANGPTL4 and BNP in combination, and hsCRP, in relation to 5-year all-cause mortality in the Argentinian cohort. The analysis uses 505 patients with known values for ANGPTL4, BNP and hsCRP.

**Figure 5:**
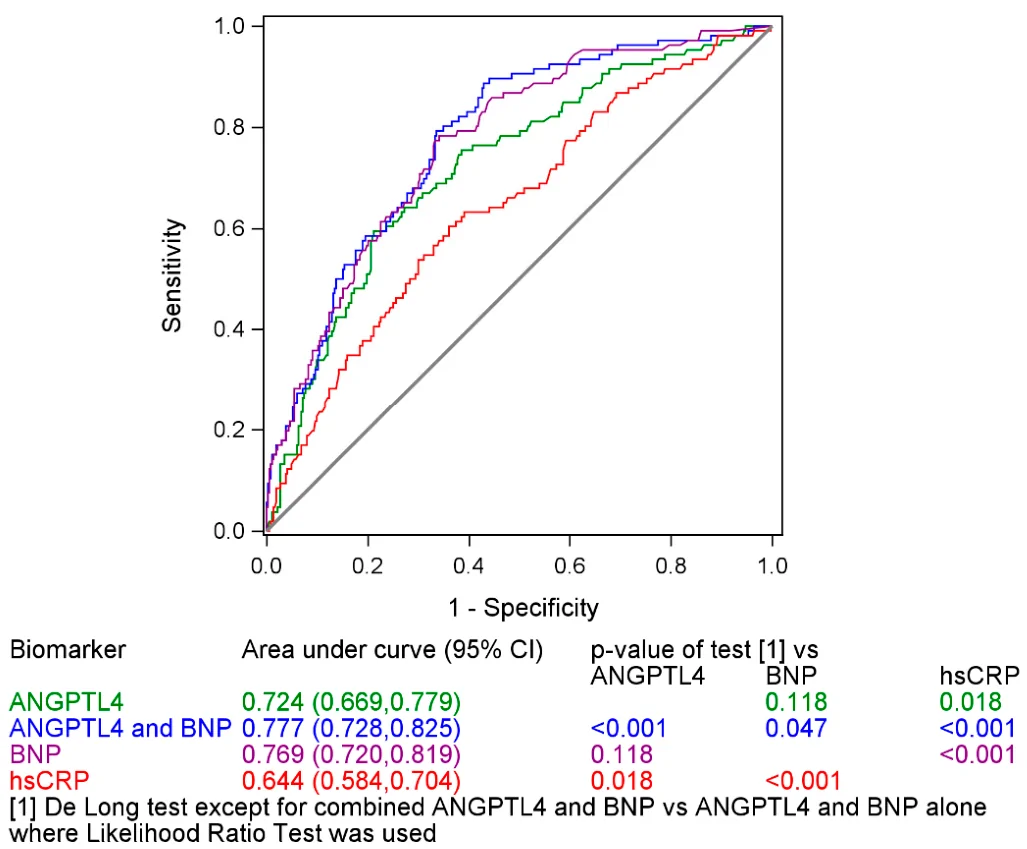
Receiver Operating Characteristic (ROC) curves and area under curve (AUC) (95% CI) values for ANGPTL4, BNP, ANGPTL4 and BNP in combination, and hsCRP, in relation to 5-year all-cause mortality in the Norwegian cohort. The analysis uses 475 patients with known values for ANGPTL4, BNP and hsCRP.

**Figure 6:**
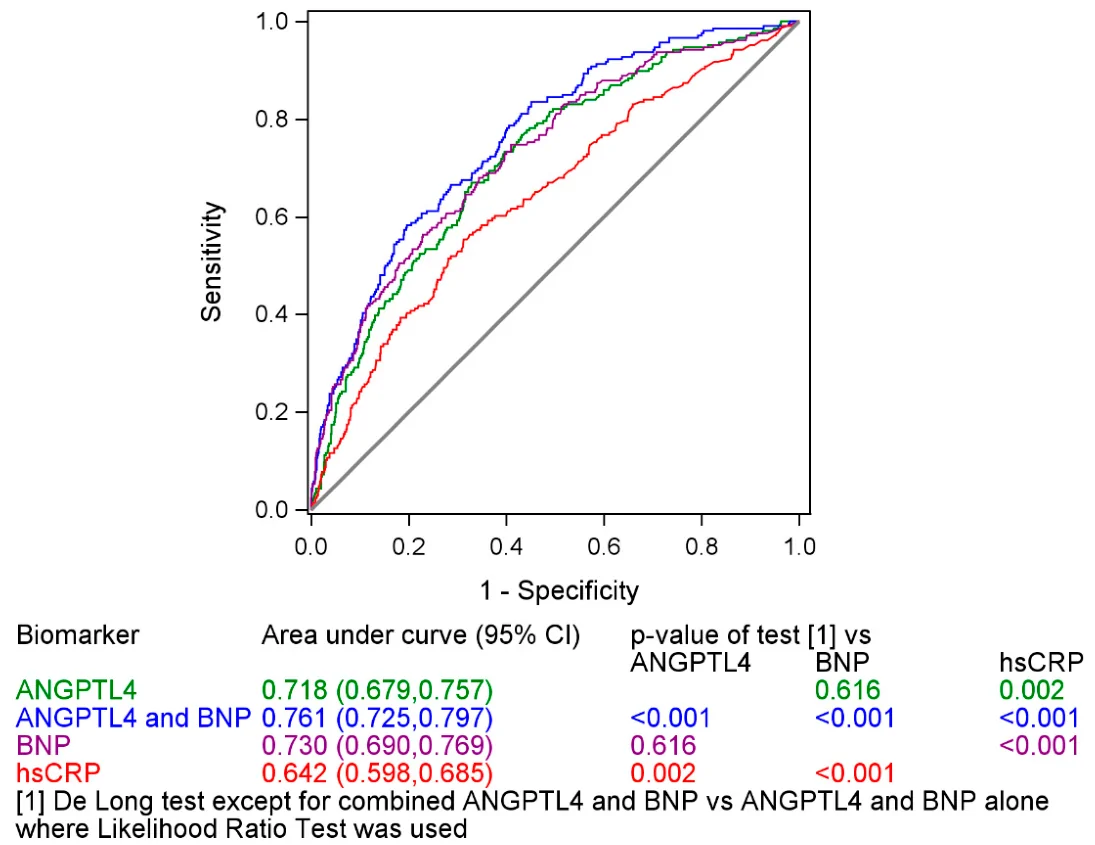
Receiver Operating Characteristic (ROC) curves and area under curve (AUC) (95% CI) values for ANGPTL4, BNP, ANGPTL4 and BNP in combination, and hsCRP, in relation to 5-year all-cause mortality in a collective assessment of the Argentinian and Norwegian cohorts matched by propensity scoring according to age, gender and the previous use of statins. The analysis uses 971 patients with known values for ANGPTL4, BNP and hsCRP.

**Figure 7:**
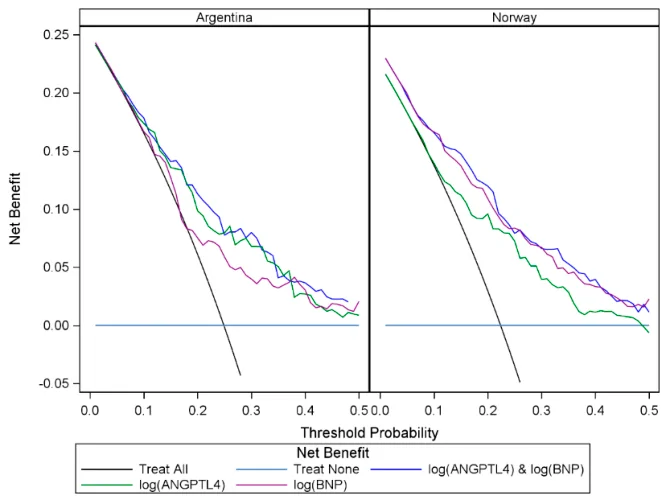
Decision curve analysis, based on univariate Kaplan–Meier estimates at five years, demonstrating the clinical utility of ANGPTL4 and BNP and their combination for prediction of 5-year all-cause mortality in the Argentinian and Norwegian cohort, respectively.

The ROC analysis of selected biomarkers in the Argentinian cohort is shown in Figure 4, for the Norwegian cohort in Figure 5, and for the combined populations in Figure 6, accompanied by a decision curve analysis in Figure 7.

In the combined population (Figure 6), the AUC for BNP was 0.730 (95%CI 0.690–0.769) as compared to 0.718 (95%CI 0.679–0.757) for ANGPTL4. For comparison, the ROC curve for hsCRP has an AUC of 0.642 (95%CI 0.598–0.685), significantly different from that of BNP only (*p* < 0.001) and ANGPTL4 only (*p* = 0.002).

### 2.6. Prognostic Significance of Combining ANGPTL4 and BNP

The likelihood ratio test was used to compare the prognostic utility of BNP and ANGPTL4 singly with their combination. ANGPTL4 and BNP did not have significantly different AUROC values for any of the populations. For Argentina, the combination of ANGPTL4 and BNP was better than BNP only (*p* = 0.004) but not ANGPTL4 (Figure 4), whereas for Norway the combination of the two biomarkers was better than ANGPTL4 (*p* = 0.011) but not BNP (Figure 5); finally, for both populations, the two biomarkers together were better than both BNP (*p* = 0.018) and ANGPTL4 (*p* = 0.001) alone (Figure 6). For comparison we show a decision curve analysis using 5-year Kaplan–Meier estimates from Cox models (Figure 7), which show less separation between the better single biomarker and the combined ANGPTL4 and BNP for each population.

### 2.7. ANGPTL4 and BNP in Relation to Cardiac Endpoints at 5 Years’ Follow-Up

In the Argentinian cohort, cardiac death was reported at 5-year follow-up, whereas all-cause mortality, MI or stroke as a composite endpoint was reported in the Norwegian cohort. Kaplan–Meier curves demonstrating the association between ANGPTL4 and BNP, respectively, and the reported cardiac/cardiovascular endpoints are displayed in Supplementary Figure S7 and Supplementary Figure S8, respectively.

Statistically significant results were obtained in the univariate and adjusted quartile analysis of the association between ANGPTL4 and cardiac death (10.1%), comparing Q4 with Q1, by which the adjusted analysis yielded an HR of 3.15 (95%CI 1.17–8.48), *p* = 0.024. Applying continuous values in univariate and multivariable analysis for the assessment of the association between **ANGPTL4** and 5-year cardiac mortality in the Argentinian population yielded an HR of 1.72 (95%CI 1.33–2.22), *p* < 0.001, and an HR of 1.32 (95%CI 0.99–1.77), *p* = 0.061, respectively, the latter reflecting a borderline association. The corresponding results related to the composite endpoint (32.8%) in the Norwegian population were HR 1.55 (95%CI 1.36–1.78), *p* < 0.001, and HR 1.07 (95%CI 0.90–1.28), *p* = 0.44.

For the Argentinian patients, univariate and multivariable assessment of the association between **BNP** and cardiac mortality yielded an HR of 2.45 (95%CI 1.83–3–28), *p* < 0.001, and an HR 2.23 (95%CI 1.63–3.06), *p*< 0.001, respectively. The corresponding results were similar for the composite endpoint in the Norwegian population with an HR of 2.09 (95%CI 1.77–2.48), *p* < 0.001, and HR 1.49 (95%CI 1.22–1.83), *p* < 0.001, respectively.

### 2.8. ANGPTL4 and Its Relation to Lipid Metabolites

In the combined populations, the Spearman correlation coefficient showed a negative correlation between log_e_ANGPTL4 and log_e_TG (rs = −0.13, *p* < 0.0001), essentially reflecting that of the Argentinian population (rs= −0.26, *p* < 0.0001), whereas only a very weak negative correlation of borderline significance was observed in the Norwegian patients (rs = −0.09, *p* = 0.049). These data are shown in a scatter plot (Supplementary Figure S9a–c).

A negative correlation between log_e_ANGPTL4 and TC was consistent for the combined (rs = −0.191, *p* < 0.0001), the Argentinian (rs = −0.13, *p* = 0.003) and the Norwegian populations (rs = −0.12, *p* = 0.01), respectively, as demonstrated in Supplementary Figure S10a–c.

A negative correlation between log_e_ANGPTL4 and LDL-C was statistically significant in the combined (rs= −0.14, *p* < 0.0001) and in the Norwegian population (rs = −0.11, *p* = 0.014), and was non-significant in the Argentinian patients (rs = −0.02, *p* = 0.694), shown in Supplementary Figure S11a–c.

As demonstrated in Supplementary Figure S12a–c, log_e_ANGPTL4 correlated negatively and weakly although significantly with HDL-C in the combined population (rs = −0.07, *p* = 0.024), but no correlation was found in the individual populations (Argentina; rs = −0.062, *p* = 0.146) (Norway; rs = −0.004, *p* = 0.937).

### 2.9. ANGPTL4 and Its Relation to Diabetes, BMI and Low-Grade Inflammation

**Figure 8:**
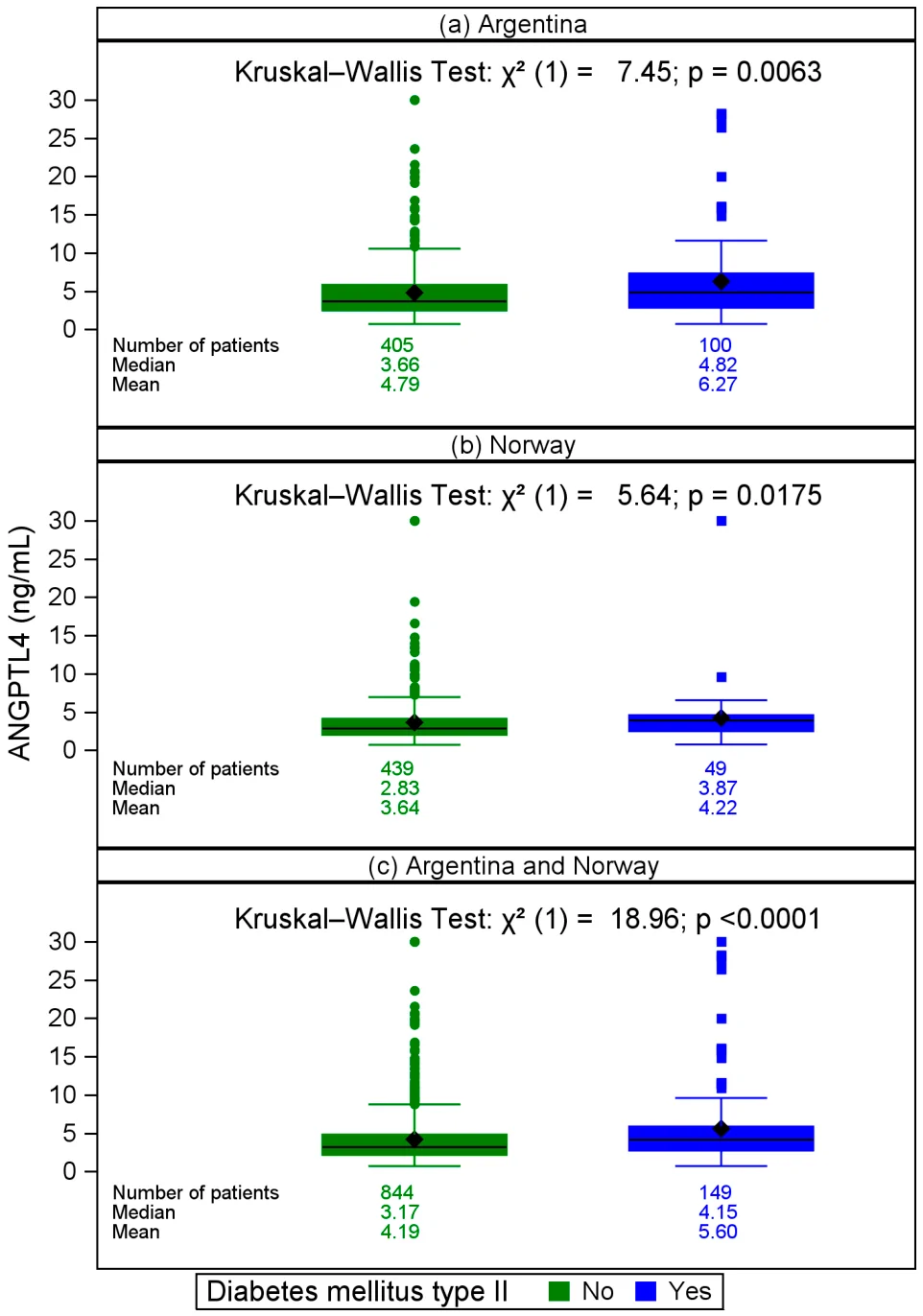
(**a**–**c**). Box plots of ANGPTL4 by history of diabetes type 2 in (**a**) the Argentinian cohort, (**b**) the Norwegian cohort, and (**c**) the combined patient population admitted with acute chest pain of suspected coronary origin and matched by propensity scoring according to age, gender and previous statin use.

**Figure 9:**
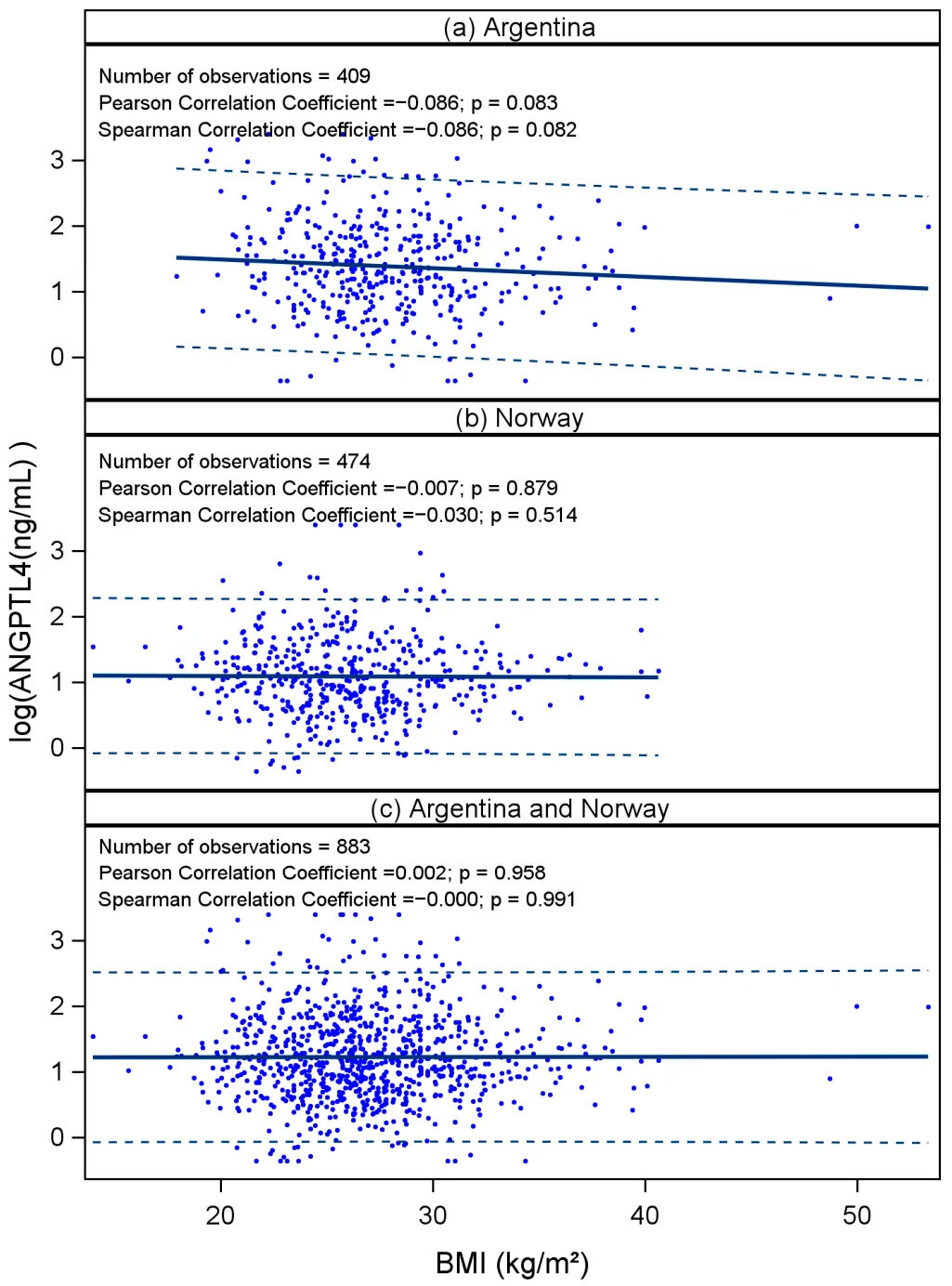
(**a**–**c**). Scatter plots demonstrating correlations between log_e_ANGPTL4 and body mass index (BMI) in (**a**) the Argentinian cohort, (**b**) the Norwegian cohort and (**c**) combined cohorts admitted with acute chest pain of suspected coronary origin and matched by propensity scoring according to age, gender and previous statin use. Solid line is fitted regression line; dotted lines are the 95% prediction interval.

**Figure 10:**
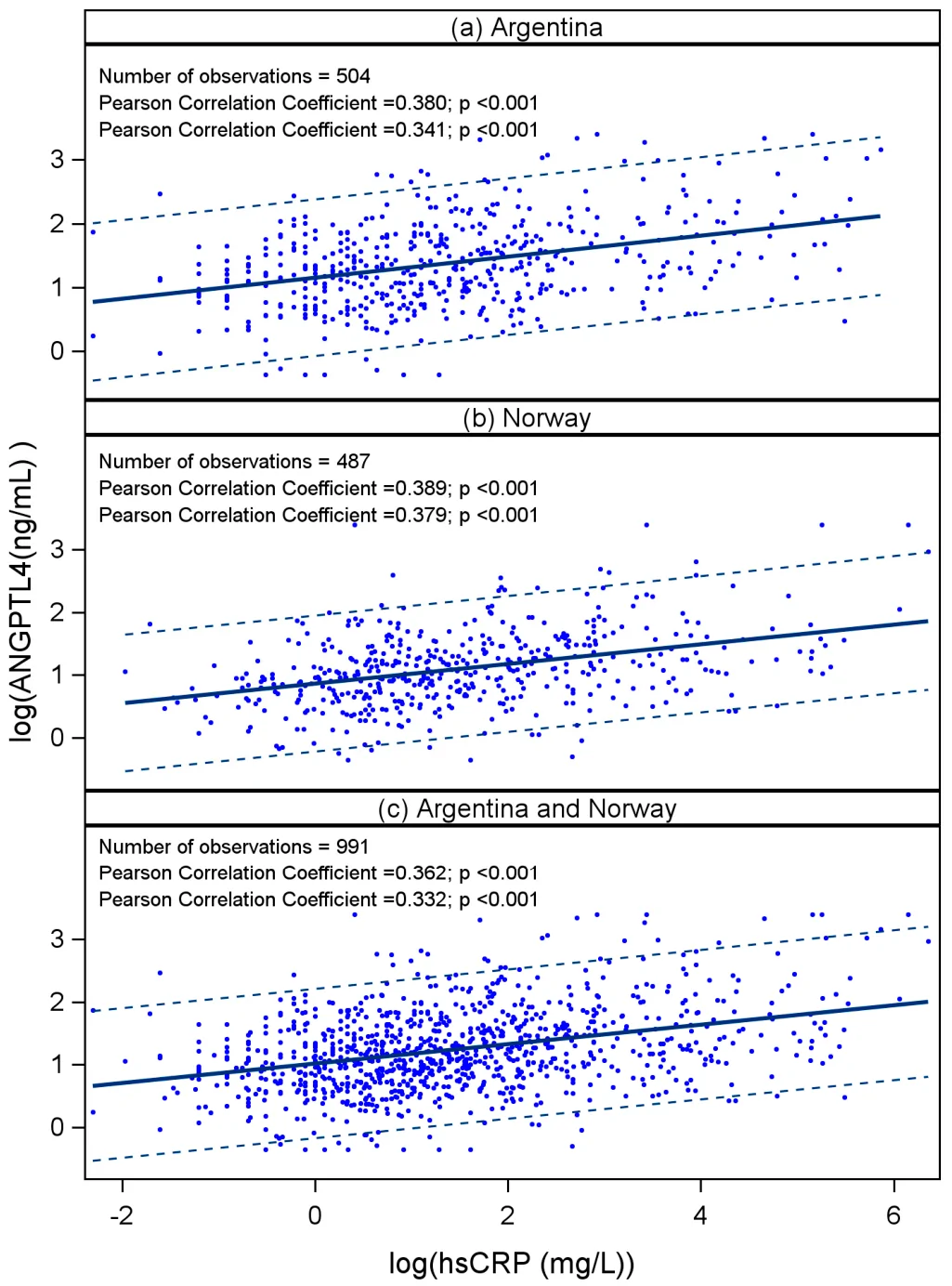
(**a**–**c**). Scatter plots demonstrating correlations between log_e_-transformed values of ANGPTL4 and high sensitivity C reactive protein (hsCRP) in (**a**) the Argentinian cohort, (**b**) the Norwegian cohort and (**c**) combined cohorts admitted with acute chest pain of suspected coronary origin and matched by propensity scoring according to age, gender and previous statin use. Solid line is fitted regression line; dotted lines are the 95% prediction interval.

Diabetics vs. non-diabetics in the Argentinian (100 vs. 405) and Norwegian cohort (49 vs. 439), as well as in the collective population (149 vs. 844), demonstrated significantly higher levels of ANGPTL4, as shown in the box plots (Figure 8a–c). No significant correlation was obtained between ANGPTL4 and BMI, as demonstrated by scatter plots (Figure 9a–c). We found, however, that log_e_ANGPTL4 was positively correlated with log_e_hsCRP in both the Argentinian (rs = 0.341, *p* < 0.001) and the Norwegian cohort (rs = 0.379, *p* < 0.001), as well as in the combined population (rs = 0.332, *p* < 0.001). Values are reported as Spearman’s correlation coefficient (Figure 10a–c).

## 3. Discussion

In this prospective study, we have compared the performance of ANGPTL4 with that of BNP as prognostic markers of 5-year all-cause mortality in an Argentinian and a Norwegian cohort admitted with chest pain of suspected coronary origin, after exactly matching by propensity scoring according to age, sex and previous use of statins. Two-year cardiac mortality and 5-year all-cause mortality were similar between the two populations, and both biomarkers were independently associated with mortality in each cohort as well as for the combined population. Similar trends were already present at 2-year follow-up. The use of ANGPTL4 as a risk marker in Argentina appears to be more strongly related to outcome compared to its use as a prognosticator in Norway, especially in men, but BNP and ANGPTL4 did not have a significantly different AUROC for any population. Yet, for Argentina the combination of ANGPTL4 and BNP was better than BNP but not for ANGPTL4, whereas the opposite was observed in the Norwegian cohort, as clearly displayed in the AUROC curves. Finally for both populations together, the two biomarkers together were better than BNP alone and ANGPTL4 alone. Our findings underscore that the combination of two biomarkers, reflecting different pathogenic pathways (myocardial remodelling and inflammation/metabolic traits, respectively), could give additional prognostic information. The results favouring the use of ANGPTL4 as a prognosticator in the Argentinian population underscore that this pattern may vary across different parts of the world, driven at least in part by metabolic differences.

It has previously been reported that ANGPTL4 may serve as a prognostic indicator of all-cause mortality in different populations including the present cohorts [16,17,22]. However, its prognostic performance compared to BNP, which has become an acclaimed prognostic indicator in coronary heart disease, has not previously been weighted in a transcontinental setting. The comparable prognostic usefulness of ANGPTL4 relative to BNP in the Argentinian population merits further attention.

At 5-year follow-up, the association between continuous values of ANGPTL4 and cardiac mortality in the Argentinian cohort did not reach statistical significance in the adjusted analysis, despite a significant adjusted difference when comparing ANGPTL4 in the highest with that of its lowest quartile. Moreover, ANGPTL4 was not associated with the composite endpoint of all-cause mortality, MI or stroke in the Norwegian cohort, whereas BNP was found to be an independent prognosticator of the cardiac/cardiovascular related endpoints in both studies. Thus, although both biomarkers serve the purpose of predicting all-cause mortality in both populations, BNP was indeed superior as a predictor of cardiac related events.

BNP is related to coronary ischemia and heart insufficiency [18,19]. In this study, the rates of congestive heart failure did not differ between the two populations and, paradoxically, we found a better correlation between ANGPTL4 and troponin-T in the Argentinian compared to the Norwegian cohort. However, as ANGPTL4 correlated better with BNP in the Norwegian cohort, this would reflect a closer association between these biomarkers in the Norwegians as compared to the Argentinians and render them less discriminative in a prognostic setting. Nonetheless, whereas BNP reflects myocardial stress, ANGPTL4 provides complementary prognostic information in MI, reflecting vascular inflammation, plaque stability, and ischemic tissue remodelling, as well as its interaction with metabolic disturbances [23].

The decision curve analysis with the combined use of ANGPTL4 and BNP demonstrated less separation between the better single biomarker and the combined ANGPTL4 and BNP. However, as these biomarkers express different pathogenic pathways, and given their discrepant weighting related to the cause of death, both should be included as predictors.

As ANGPTL4 seems to challenge BNP in the Argentinian population, we explored its relation to TG. ANGPTL4 acts as a critical regulator of lipid metabolism by inhibiting LPL and significantly impacts on the processing and distribution of TG and cholesterol [24]. Decreased ANGPTL4 function is associated with a beneficial effect on lipid parameters and risk of metabolic dysfunction and cardiovascular disease [1,5,24]. Herein, ANGPTL4 and TG levels were significantly higher in the Argentinian than in the Norwegian population, whereas the opposite relationship was noted for TC, HDL-C and LDL-C. The Argentinians were recruited from an inland area (Salta) of Argentina, where ingestion of red meat is high and fish consumption low [25], whereas the Norwegian study population was recruited from a coastal area (Rogaland) of Norway, where fish consumption is high [26]. It is known that omega-3 FA, with fish as an important source, reduces very-low-density lipoprotein production by inhibiting hepatic lipogenesis and promoting fatty acid β-oxidation [7], whereas organ-specific LPL activity in tissues such as heart and skeletal muscle is regulated through a feedback mechanism involving ANGPTL4. An influx of TG-derived FAs increases the expression of ANGPTL4, preventing further uptake of FAs [14,27], and this feedback mechanism on ANGPTL4 is augmented by long-chain polyunsaturated FAs, resulting in a locally reduced breakdown of TG [7]. However, the net systemic effect of fish-derived FAs is a reduction in triglycerides and ANGPTL4. Thus, a low consumption of fish and products derived from fish could potentially lead to higher levels of ANGPTL4 and TG, in line with what was observed in the Argentinians compared to the Norwegian population.

In the combined population we observed a highly significant but weak negative correlation between ANGPTL4 and TG, TC and LDL-C, while a correlation was inconspicuous for HDL-C. ANGPTL4 was more inversely correlated with both TG and TC in the Argentinian compared to the Norwegian population and did not correlate with either LDL-C or HDL-C in the Argentinians. While Mehta et al. [28], in an investigation including 1770 subjects, found a negative correlation between ANGPTL4 and LDL-C and HDL-C—as in our study—their observation of a weak but significant positive correlation with TG (r = 0.081, *p* = 0.0008) was opposed to our results and has not been corroborated by other studies that report no or a weak negative correlation [14,15,29,30]. Interestingly, findings by Nilsson et al. [31], that the prime site of ANGPTL4 action may be the subendothelial compartment, could influence the lack of a positive correlation between circulating ANGPTL4 and TG.

In the study by Mehta et al. [28], a stratified analysis showed that among those without diabetes mellitus, there was no significant association between ANGPTL4 and HDL-C, LDL-C, TC, or TG. In subjects with diabetes, the authors observed a significant negative association between ANGPTL4 and HDL-C, together with a positive association with TG. The authors reported significantly higher ANGPTL4 levels among individuals with type 2 diabetes compared with non-diabetics, in line with our findings and in contrast to the opposite findings by Swarnakar et al. [32]. Taken together, the influence of ANGPTL4 on lipid metabolism is rather complex.

In addition to its association with lipid and glucose metabolism [28,30], ANGPTL4 is also associated with inflammation [33]. In the present study, there was a significant and moderate positive correlation between ANGPTL4 and CRP in both patient cohorts, corroborating the findings by Tjeerdema et al. [33]. Its ability to mirror both lipid metabolism and inflammation, the two major pathogenic and interacting pathways in atherogenesis, could contribute to its utility as a prognostic biomarker in atherosclerotic disorders [34].

The prognostic performance of ANGPTL4 seems to be closely related to metabolic and inflammatory drivers of all-cause mortality, with a trend towards cardiac death, whereas BNP is more strongly associated with cardiac death and non-fatal cardiovascular events.

### Limitations

The target population for this study was patients admitted with chest pain of clinically suspected coronary origin. Not only the acute MI population, but also a large number of non-AMI patients, presented with a history of established coronary heart disease, and a considerable proportion of those who were not diagnosed as having sustained an AMI presented a minor troponin release as judged by retrospective measurements of high sensitivity TnT. Nevertheless, the lack of a definite coronary-related diagnosis in patients without an increase in TnT is a study limitation. Time from symptom onset was not accounted for, which may have influenced the levels of biomarkers, especially in the AMI patients, but would be considered of less importance in non-AMI patients. Blood sampling for the measurement of biomarkers was limited to one draw. We did not measure left ventricular ejection fraction, but used BNP as a surrogate, and the between-country difference in this parameter was nonsignificant. The medical management of patients in the time periods during which the studies were executed was very similar for the two countries. Index revascularizations were not included in our analysis, as they were not fully accounted for in the Argentinian population. Due to incomplete information on BMI, we did not adjust for this covariable in the individual populations, whereas other potential confounders were accounted for all through the entire study. Furthermore, as this is an exploratory study, we did not adjust for multiplicity.

## 4. Materials and Methods

Two patient cohorts with clinically suspected acute coronary syndrome (ACS) were recruited consecutively based on the same protocol in Argentina [ClinicalTrials.gov NCT01377402] and in Norway [ClinicalTrials.gov NCT00521976], respectively, and were prospectively followed up for 5 years employing the same case report form (CRF) [17]. Exclusion criteria were age < 18 years, unwillingness or incapacity to provide informed consent, and prior inclusion in the same study. The Norwegian population was recruited at the Emergency Department of Stavanger University Hospital, Norway, from November 2002 to September 2003, and consisted of 871 patients, whereas the Argentinian population was recruited at nine hospitals in Salta, Argentina, from December 2005 to January 2009, and consisted of 982 patients.

To assess the transcontinental prognostic utility of ANGPTL4, group similarity was optimized by matching the two populations exactly by propensity scoring according to age, sex and previous use of statins, leaving 507 patients in each population. BNP was used as the reference prognostic indicator. Clinical status was obtained from hospital and public registries and by telephonic interviews at 30 days and 6, 12 and 24 months, and from supportive entities, if needed. Later follow-up information on survival status was provided from death registries.

Baseline clinical and laboratory data were collected on admission and during hospitalization. ACS included patients with intermediate troponin-T (TnT) values, non-ST segment elevation myocardial infarction (NSTEMI) and ST-segment elevation myocardial infarction (STEMI). Due to the use of different generations of TnT assays, we used a TnT cut-off value of 50 ng/L in the Norwegian population and 30 ng/L in the Argentinian population, respectively, for the definition of an acute myocardial infarction (MI) [34]. In a subsample of patients with TnT levels ≤ 10 ng/L, approximately 70% had an elevated high-sensitivity troponin-T (hsTnT) of ≥5 ng/L [35].

### 4.1. Ethics

The Norwegian study was approved by the Regional Board of Research Ethics and by the National Health Authorities. The Argentinian study was approved by the Ethics Committee at the Board of Medical School of Salta, Argentina. Both studies were conducted in accordance with the Helsinki declaration of 1971, as revised in 1983.

### 4.2. Laboratory Tests

Immediately following admission, blood was harvested from an antecubital vein. A second blood sample for repeat TnT was obtained after 6–7 h. TnT, creatinine, glucose and lipids [triglycerides (TGs), total cholesterol (TC) and high-density lipoprotein cholesterol (HDL-C)] were measured locally immediately after centrifugation by the Central Laboratory in Stavanger and by a central laboratory unit with a dedicated biochemical team shared by the hospitals in Salta. We used the Friedewald equation for the assessment of low-density lipoprotein cholesterol (LDL-C), excluding TG > 4.5 mmol/L from the formula. Aliquots of ethylenediaminetetraacetic acid (EDTA) plasma, citrated plasma, and serum were stored at −80 °C for later measurements.

*ANGPTL4* was measured in EDTA plasma by enzyme-linked immunosorbent assay (ELISA) technique. Among the total population, citrated plasma was used in 65 patients when EDTA plasma was lacking. ANGPTL4 was analyzed in duplicate with antibodies designed to detect the full length ANGPTL4 protein and the C-terminal fragment but not the N-terminal fragment (Cat# DY3485 from R&D Systems, Stillwater, MN, USA) in a 384-well format employing a combination of a SELMA (Jena, Germany) pipetting robot and a BioTek (Winooski, VT, USA) dispenser/washer. Absorption was read at 450 nm with wavelength correction set to 540 nm using an ELISA plate reader (BioTek). Intra- and inter-assay coefficients were <10%. Measurements in EDTA and citrated plasma did not differ significantly [17].

*BNP* was measured using Microparticle Enzyme Immunoassay Abbott AxSYM (Abbott Laboratories, Abbott Park, IL, USA).

ANGPTL4 and BNP samples from both populations were measured in the same run.

### 4.3. Statistics

Propensity scoring was used to match patients in the Norwegian and Argentinian populations. Only patients in the unwidened common support region, defined by the logit of the propensity score, were eligible for matching. Randomly ordered Norwegian patients were matched, without a calliper, exactly 1:1 on age (rounded to the nearest integer), sex and prior statin use with their nearest neighbour.

Descriptive statistics are presented as medians with interquartile ranges (25th–75th percentiles) for continuous data and as numbers and percentages for categorical data. Differences in baseline characteristics were assessed by the Kruskal–Wallis test for continuous data and the Chi-squared test for categorical data. Due to a skewed distribution, Pearson’s and Spearman’s rank correlation coefficients were calculated between the log_e_-transformed values of ANGPTL4 and lipids.

No patients were lost to follow-up, but two Argentinian patients had unknown time to death. Patients without an event were censored at five years. The Kaplan–Meier product limits were used for plotting the times to events, and the log-rank test was used to test for the equality of the survival curves. Cox regression models were fitted for each endpoint and biomarker combination showing Wald’s Hazard ratio (HR), 95% confidence intervals and a two-sided *p*-value when testing against a value of 1. The first model used the biomarker quartile with quartile-1 as the reference level. For the second model, the log_e_-transformed value of biomarkers were normalized by dividing by their standard deviation (SD). Results are shown separately for the univariate (UVA) and multivariable analysis (MVA). The multivariable model included potential covariables [shown by an asterisk in the baseline table (Table 1)] selected using a stepwise algorithm adding to the model containing ANGPTL4, applying a *p*-value threshold of 0.05 for inclusion and 0.10 for removal. Receiver Operating Characteristic (ROC) curves were plotted to assess the prognostic performance of the biomarkers by their area under curve (AUC). De Long’s test was used to determine differences between AUROCs for pairs of biomarkers. The likelihood ratio test was used to test between the combination of ANGPTL4 and BNP against each of these individual biomarkers. The decision curve analysis used the 5-year Kaplan–Meier estimates from the respective univariate or bivariate Cox model.

Statistics were performed using the statistical package SPSS version 25 (IBM Corp. Armonk, NY, USA). All tests were 2-sided with a significance level of 5% without multiplicity adjustment.

## 5. Conclusions

In conclusion, the use of ANGPTL4 as a risk marker in Argentina appears to be more strongly related to outcome compared to its use as a prognosticator in Norway, especially in men, but BNP and ANGPTL4 did not have a significantly different AUROC for any population. For Argentina the combination of ANGPTL4 and BNP was better than BNP but not for ANGPTL4, whereas the opposite was observed in the Norwegian cohort. Finally, for both populations collectively, the two biomarkers together were better than both BNP and ANGPTL4 alone. Our findings suggest a complex interaction between ANGPLT4 and metabolic parameters that also may differ between different continents.

## Figures and Tables

**Table 1:** Baseline features in the Argentinian and the Norwegian cohorts admitted with acute chest pain of suspected coronary origin, matched by propensity scoring according to age, gender and previous statin treatment.

Characteristics	Total n = 994	Argentina n = 506	Norway n = 488	***p*** **-Value**
Demographics				
Age (years) *	66.2 (54.5–76.0)	67.0 (55.0–76.0)	66.1 (54.3–76.1)	0.714
Males n (%) *	624 (62.78)	316 (62.45)	308 (63.11)	0.829
Biomarkers				
ANGPTL4 (ng/mL)	3.3 (2.2–5.1)	3.7 (2.5–6.1)	2.9 (2.0–4.3)	<0.001
BNP (ng/L) *	75.0 (30.5–217.1)	86.7 (35.3–207.0)	64.0 (24.0–235.0)	0.055
hs CRP (mg/L) *	3.4 (1.4–10.0)	3.2 (1.3–8.7)	3.7 (1.7–12.2)	0.041
eGFR (ml/min/1.73 m^2^) *	72.7 (57.8–89.3)	80.4 (62.0–97.7)	67.6 (55.1–78.2)	<0.001
TC (mmol/L) *	5.0 (4.3–5.8)	4.6 (3.9–5.4)	5.5 (4.7–6.3)	<0.001
Acute MI # n (%)	408 (41.09)	178 (35.25)	230 (47.13)	<0.001
TnT ng/L ranges				<0.001
TnT release ≤ 10 ng/L n (%)	647 (65.16)	365 (72.28)	282 (57.79)	
TnT release > 10 and < 30 ng/L n (%)	71 (7.15)	31 (6.14)	40 (8.20)	
TnT release ≥ 30 and < 50 ng/L n (%)	46 (4.63)	20 (3.96)	26 (5.33)	
TnT release ≥ 50 ng/L n (%)	229 (23.06)	89 (17.62)	140 (28.69)	
Index STEMI n (%)	170 (17.19)	77 (15.37)	93 (19.06)	0.124
Risk factors				
Smoking *				<0.001
Ex-smoker n (%)	274 (27.57)	116 (22.92)	158 (32.38)	
Current smoker n (%)	450 (45.27)	291 (57.51)	159 (32.58)	
Never smoked n (%)	270 (27.16)	99 (19.57)	171 (35.04)	
Hypertension n (%) *	526 (52.92)	346 (68.38)	180 (36.89)	<0.001
Diabetes mellitus type I n (%) *	10 (1.01)	8 (1.58)	2 (0.41)	0.064
Diabetes mellitus type II n (%) *	149 (15.01)	100 (19.80)	49 (10.04)	<0.001
TC > 6.5 mmol/L n (%) *	128 (12.90)	25 (4.96)	103 (21.11)	<0.001
BMI (kg/m^2^) ‡	26.5 (24.1–29.4)	27.3 (25.1–30.1)	25.7 (23.4–28.4)	<0.001
History of heart disease				
Angina pectoris n (%) *	291 (29.28)	123 (24.31)	168 (34.43)	<0.001
Previous MI n (%) *	149 (14.99)	59 (11.66)	90 (18.44)	0.003
Previous CABG n (%) *	52 (5.23)	29 (5.73)	23 (4.71)	0.471
Previous PCI n (%) *	90 (9.05)	64 (12.65)	26 (5.33)	<0.001
Heart failure n (%) *	172 (17.30)	94 (18.58)	78 (15.98)	0.280
Treatment prior to admission				
ACEI or ARB n (%) *	356 (35.81)	227 (44.86)	129 (26.43)	<0.001
Beta-blockers n (%) *	264 (26.56)	149 (29.45)	115 (23.57)	0.036
Statins n (%) *	153 (15.39)	78 (15.42)	75 (15.37)	0.984
Acetylsalicylic acid n (%) *	314 (31.59)	183 (36.17)	131 (26.84)	0.002

Data are presented as median (interquartile range) or numbers (%). Abbreviations: ANGPTL4, Angiopoietin-like 4. BNP, B-type natriuretic peptide. hs-CRP, high-sensitivity C-reactive protein. eGFR, estimated glomerular filtration rate. TC, total cholesterol. TnT, troponin-T. MI, myocardial infarction. CABG, coronary artery bypass grafting. PCI, percutaneous coronary intervention. STEMI, ST-Elevation Myocardial Infarction. ACEI, angiotensin-converting-enzyme inhibitor. ARB, angiotensin receptor blocker. # For the diagnosis of an acute myocardial infarction, we applied a TnT cut-off value of 30 ng/L for the Argentinian population and 50 ng/L for the Norwegian population, as 2 different TnT assays were used. ‡ BMI was not adjusted for, as there were 97 missing values in the Argentinian and 10 missing values in the Norwegian population, respectively. *p*-values represent differences between the Argentinian and the Norwegian cohort. * The asterisk indicates potential covariates adjusted for in the multivariable analysis. Those retained in the model for the combined cohorts were as follows: age over 10 years, chronic heart failure, BNP quartile, logeGFR, CRP quartile and TnT > 10 ng/L, in that order. Age over 10 years and chronic heart failure were common for both cohorts.

## Data Availability

The original contributions presented in this study are included in the article/Supplementary Material. Further inquiries can be directed to the corresponding author.

## Supplementary Materials

The following supporting information can be downloaded at: https://www.mdpi.com/article/10.3390/ijms27219966/s1.
