# Supplementary material for: Angiopoietin-like 4 (ANGPTL4) Protein and Its Relation to Lipids Challenges the Prognostic Performance of B-Type Natriuretic Peptide (BNP) Transcontinentally in Patients with Suspected Coronary Chest Pain, Suggesting Their Combined Use in Risk Prediction
Source: Int J Mol Sci. 2026 Nov 6;27(21):9966. doi: 10.3390/ijms27219966 (PMC13763694; doi:10.3390/ijms27219966)

Table S1. Baseline clinical features according to quartiles of angiopoietin-like 4 (ANGPTL4) in the Argentinian population

Characteristics	Total<br>n = 506	Quartile 1<br>n = 126	Quartile 2<br>n = 127	Quartile 3<br>n = 127	Quartile 4<br>n = 126	p-Value
Demographics						
Age (years) *	67.0 (55.0 - 76.0)	60.5 (51.0 - 72.0)	62.0 (52.0 - 74.0)	68.0 (57.0 - 76.0)	74.0 (66.0 - 80.0)	<0.001
Males n(%) *	316 (62.45)	79 (62.70)	95 (74.80)	80 (62.99)	62 (49.21)	<0.001
Biomarkers						
ANGPTL4 (ng/mL)	3.7 (2.5 - 6.1)	1.9 (1.6 - 2.2)	3.1 (2.8 - 3.4)	4.7 (4.1 - 5.3)	8.3 (7.1 - 11.6)	<0.001
BNP (ng/L) *	86.7 (35.3 - 207.0)	59.6 (26.7 - 116.6)	57.5 (28.8 - 147.2)	104.9 (40.0 - 233.3)	143.6 (66.2 - 394.6)	<0.001
hs CRP (mg/L) *	3.2 (1.3 - 8.7)	1.9 (0.9 - 4.6)	2.7 (1.1 - 6.1)	3.8 (1.4 - 9.5)	6.1 (2.8 - 29.9)	<0.001
eGFR (ml/min/1.73 m2) *	80.4 (62.0 - 97.7)	87.4 (74.3 - 98.6)	88.3 (72.1 - 103.2)	77.6 (61.2 - 97.2)	60.0 (42.2 - 84.4)	<0.001
TC (mmol/L) *	4.6 (3.9 - 5.4)	4.8 (4.2 - 5.7)	4.6 (4.0 - 5.3)	4.5 (3.9 - 5.4)	4.4 (3.6 - 5.3)	0.026
Acute MI # n(%)	178 (35.25)	25 (20.00)	38 (29.92)	53 (41.73)	62 (49.21)	<0.001
TnT ng/L ranges						<0.001
TnT release ≤ 10 ng/L n(%)	365 (72.28)	107 (85.60)	93 (73.23)	85 (66.93)	80 (63.49)	
TnT release > 10 and < 30 ng/L n(%)	31 (6.14)	8 (6.40)	9 (7.09)	11 (8.66)	3 (2.38)	
TnT release ≥ 30 and < 50 ng/L n(%)	20 (3.96)	4 (3.20)	5 (3.94)	5 (3.94)	6 (4.76)	
TnT release ≥ 50 ng/L n(%)	89 (17.62)	6 (4.80)	20 (15.75)	26 (20.47)	37 (29.37)	
Index STEMI n(%)	77 (15.37)	9 (7.20)	20 (16.00)	28 (22.05)	20 (16.13)	0.013
Risk factors						
Smoking *						0.006
Ex--smoker n(%)	116 (22.92)	35 (27.78)	40 (31.50)	24 (18.90)	17 (13.49)	
Current smoker n(%)	291 (57.51)	71 (56.35)	58 (45.67)	77 (60.63)	85 (67.46)	
Never smoked n(%)	99 (19.57)	20 (15.87)	29 (22.83)	26 (20.47)	24 (19.05)	
Hypertension n(%) *	346 (68.38)	81 (64.29)	86 (67.72)	89 (70.08)	90 (71.43)	0.634
Diabetes mellitus type I n(%) *	8 (1.58)	0 (0.00)	2 (1.57)	3 (2.36)	3 (2.38)	0.389
Diabetes mellitus type II n(%) *	100 (19.80)	17 (13.60)	23 (18.11)	30 (23.62)	30 (23.81)	0.127
TC > 6.5 mmol/L n(%) *	25 (4.96)	6 (4.76)	8 (6.35)	5 (3.94)	6 (4.80)	0.846
BMI (kg/m2) ‡	27.3 (25.1 - 30.1)	27.4 (25.4 - 30.7)	27.5 (25.6 - 30.1)	27.4 (25.3 - 30.1)	26.9 (24.3 - 29.5)	0.436

History of heart disease						
Angina pectoris n(%) *	123 (24.31)	30 (23.81)	28 (22.05)	33 (25.98)	32 (25.40)	0.886
Previous MI n(%) *	59 (11.66)	15 (11.90)	6 (4.72)	20 (15.75)	18 (14.29)	0.031
Previous CABG n(%) *	29 (5.73)	4 (3.17)	8 (6.30)	8 (6.30)	9 (7.14)	0.544
Previous PCI n(%) *	64 (12.65)	22 (17.46)	14 (11.02)	16 (12.60)	12 (9.52)	0.255
Heart failure n(%) *	94 (18.58)	20 (15.87)	21 (16.54)	23 (18.11)	30 (23.81)	0.354
Treatment prior to admission						
ACEI or ARB n(%) *	227 (44.86)	50 (39.68)	53 (41.73)	56 (44.09)	68 (53.97)	0.106
Beta-blockers n(%) *	149 (29.45)	41 (32.54)	30 (23.62)	40 (31.50)	38 (30.16)	0.401
Statins n(%) *	78 (15.42)	20 (15.87)	15 (11.81)	20 (15.75)	23 (18.25)	0.557
Acetylsalicylic acid0n(%) *	183 (36.17)	47 (37.30)	37 (29.13)	45 (35.43)	54 (42.86)	0.153

Data are presented as median (interquartile range) or numbers (%). Abbreviations: ANGPTL4, Angiopoietin-like 4. BNP, B-type natriuretic peptide. hs- CRP, high-sensitivity C-reactive protein. eGFR, estimated glomerular filtration rate. TC, total cholesterol. TnT, troponin-T. MI, myocardial infarction CABG, coronary artery bypass grafting. PCI, percutaneous coronary intervention. STEMI, ST-Elevation Myocardial Infarction. ACEI, angiotensin-converting-enzyme inhibitor. ARB, angiotensin receptor blocker. For the diagnosis of an acute myocardial infarction, we applied a TnT cut-off value of 30 ng/L for the Argentinian population. *The asterisk indicates covariates adjusted for in the multivariable analysis. ‡BMI was not adjusted for, as there were 97 missing values.

Table S2. Baseline clinical features according to quartiles of angiopoietin-like 4 (ANGPTL4) in the Norwegian population

Characteristics	Total n = 488	Quartile 1 n = 122	Quartile 2 n = 122	Quartile 3 n = 122	Quartile 4 n = 122	p-Value
Demographics						
Age (years) *	66.1 (54.3 - 76.1)	56.3 (48.7 - 68.9)	62.9 (54.0 - 74.2)	69.0 (58.0 - 78.3)	74.7 (65.1 - 82.7)	<0.001
Males n(%) *	308 (63.11)	86 (70.49)	75 (61.48)	67 (54.92)	80 (65.57)	0.077
Biomarkers						
ANGPTL4 (ng/mL)	2.9 (2.0 - 4.3)	1.6 (1.3 - 1.8)	2.5 (2.2 - 2.6)	3.4 (3.2 - 3.7)	5.8 (4.8 - 7.4)	<0.001
BNP (ng/L) *	64.0 (24.0 - 235.0)	28.0 (12.0 - 69.0)	48.0 (21.0 - 123.0)	90.0 (31.0 - 284.0)	244.0 (63.0 - 618.0)	<0.001
hs CRP (mg/L) *	3.7 (1.7 - 12.2)	2.0 (1.1 - 4.8)	2.6 (1.2 - 5.8)	4.7 (2.4 - 14.8)	9.0 (2.5 - 20.0)	<0.001
eGFR (ml/min/1.73 m2) *	67.6 (55.1 - 78.2)	75.6 (67.1 - 87.7)	69.8 (61.5 - 80.1)	64.7 (53.4 - 75.8)	53.3 (37.8 - 69.0)	<0.001
TC (mmol/L) *	5.5 (4.7 - 6.3)	5.5 (4.8 - 6.3)	5.7 (5.0 - 6.7)	5.6 (4.9 - 6.3)	5.0 (4.5 - 6.0)	0.002
Acute MI # n(%)	230 (47.13)	50 (40.98)	53 (43.44)	65 (53.28)	62 (50.82)	0.169
TnT ng/L ranges						0.351
TnT release ≤ 10 ng/L n(%)	282 (57.79)	78 (63.93)	74 (60.66)	64 (52.46)	66 (54.10)	
TnT release > 10 and < 30 ng/L n(%)	40 (8.20)	6 (4.92)	9 (7.38)	15 (12.30)	10 (8.20)	
TnT release ≥ 30 and < 50 ng/L n(%)	26 (5.33)	8 (6.56)	5 (4.10)	4 (3.28)	9 (7.38)	
TnT release ≥ 50 ng/L n(%)	140 (28.69)	30 (24.59)	34 (27.87)	39 (31.97)	37 (30.33)	
Index STEMI n(%)	93 (19.06)	23 (18.85)	20 (16.39)	24 (19.67)	26 (21.31)	0.802
Risk factors						
Smoking *						0.032
Ex-smoker n(%)	158 (32.38)	52 (42.62)	39 (31.97)	38 (31.15)	29 (23.77)	
Current smoker n(%)	159 (32.58)	36 (29.51)	45 (36.89)	40 (32.79)	38 (31.15)	
Never smoked n(%)	171 (35.04)	34 (27.87)	38 (31.15)	44 (36.07)	55 (45.08)	
Hypertension n(%) *	180 (36.89)	33 (27.05)	40 (32.79)	51 (41.80)	56 (45.90)	0.009
Diabetes mellitus type I n(%) *	2 (0.41)	1 (0.82)	0 (0.00)	0 (0.00)	1 (0.82)	0.571
Diabetes mellitus type II n(%) *	49 (10.04)	6 (4.92)	11 (9.02)	13 (10.66)	19 (15.57)	0.049
TC > 6.5 mmol/L n(%) *	103 (21.11)	25 (20.49)	33 (27.05)	28 (22.95)	17 (13.93)	0.085
BMI (kg/m2) ‡	25.7 (23.4 - 28.4)	25.6 (23.7 - 28.1)	26.1 (23.9 - 29.1)	25.7 (22.7 - 29.1)	25.4 (22.9 - 28.1)	0.550

History of heart disease						
Angina pectoris n(%) *	168 (34.43)	39 (31.97)	39 (31.97)	42 (34.43)	48 (39.34)	0.581
Previous MI n(%) *	90 (18.44)	13 (10.66)	19 (15.57)	23 (18.85)	35 (28.69)	0.003
Previous CABG n(%) *	23 (4.71)	5 (4.10)	5 (4.10)	5 (4.10)	8 (6.56)	0.745
Previous PCI n(%) *	26 (5.33)	9 (7.38)	6 (4.92)	6 (4.92)	5 (4.10)	0.691
Heart failure n(%) *	78 (15.98)	4 (3.28)	11 (9.02)	23 (18.85)	40 (32.79)	<0.001
Treatment prior to admission						
ACEI or ARB n(%) *	129 (26.43)	16 (13.11)	30 (24.59)	37 (30.33)	46 (37.70)	<0.001
Beta-blockers n(%) *	115 (23.57)	26 (21.31)	27 (22.13)	25 (20.49)	37 (30.33)	0.239
Statins n(%) *	75 (15.37)	21 (17.21)	19 (15.57)	15 (12.30)	20 (16.39)	0.727
Acetylsalicylic acid0n(%) *	131 (26.84)	34 (27.87)	32 (26.23)	29 (23.77)	36 (29.51)	0.773

Data are presented as median (interquartile range) or numbers (%). Abbreviations: ANGPTL4, Angiopoietin-like 4. BNP, B-type natriuretic peptide. hs- CRP, high-sensitivity C-reactive protein. eGFR, estimated glomerular filtration rate. TC, total cholesterol. TnT, troponin-T. MI, myocardial infarction CABG, coronary artery bypass grafting. PCI, percutaneous coronary intervention. STEMI, ST-Elevation Myocardial Infarction. ACEI, angiotensin-converting-enzyme inhibitor. ARB, angiotensin receptor blocker. For the diagnosis of an acute myocardial infarction, we applied a TnT cut-off value of 30 ng/L for the Argentinian population. *The asterisk indicates covariates adjusted for in the multivariable analysis. ‡BMI was not adjusted for, as there were 97 missing values

.

Figure S1. Box plots comparing the angiopoietin-like 4 (ANGPTL4) and B-type natriuretic peptide (BNP) levels in the Argentinian and the Norwegian population, matched by propensity scoring according to age, gender and statin use.

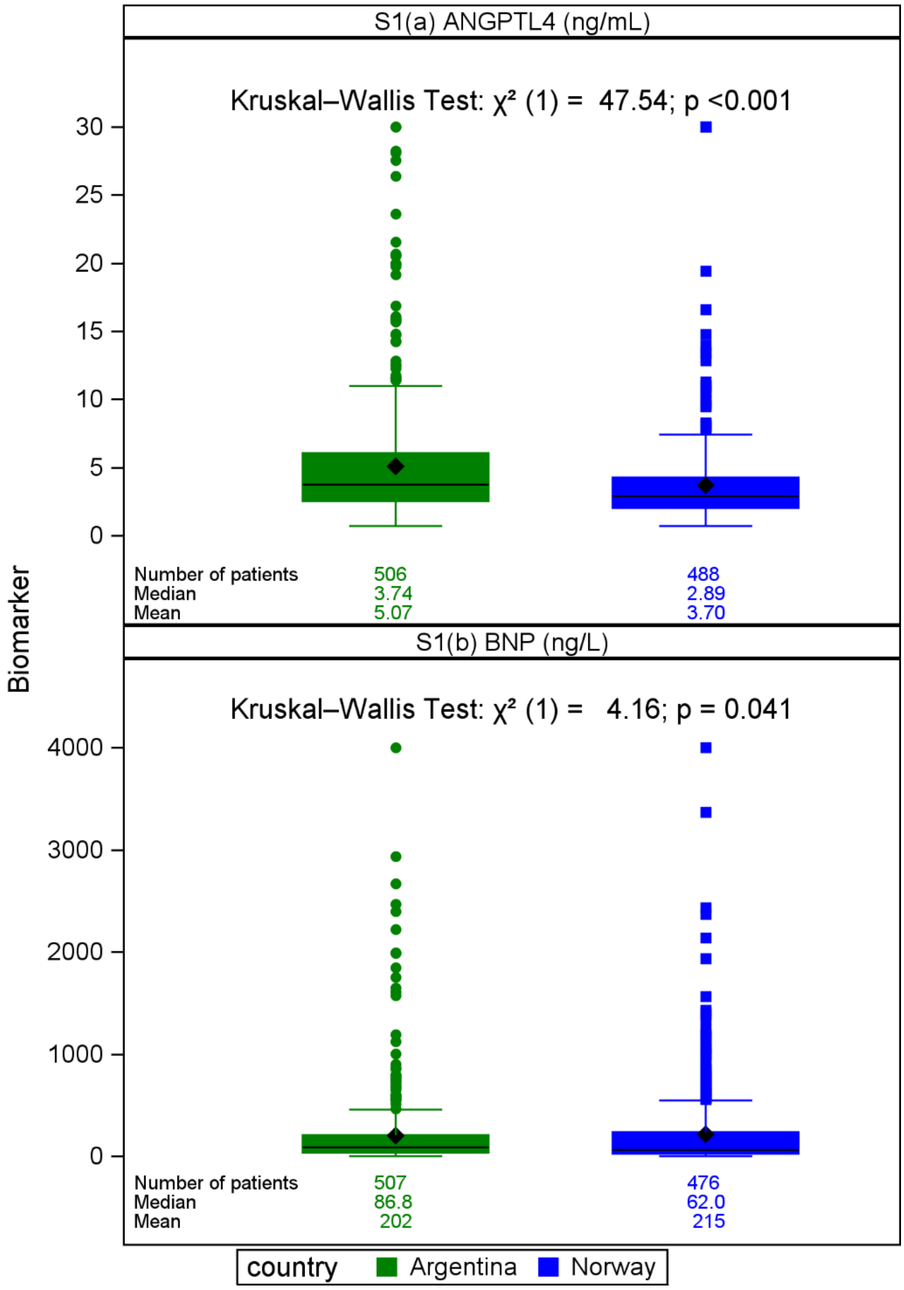

Figure S2a-b-c-d shows the box plots comparing the levels of a) triglycerides (TG), b) total cholesterol (TC), c) low density lipoprotein-cholesterol (LDL-C) and d) high density lipoprotein cholesterol (HDL-C) in the Argentinian versus the Norwegian population, matched by propensity scoring according to age, gender and statin use.

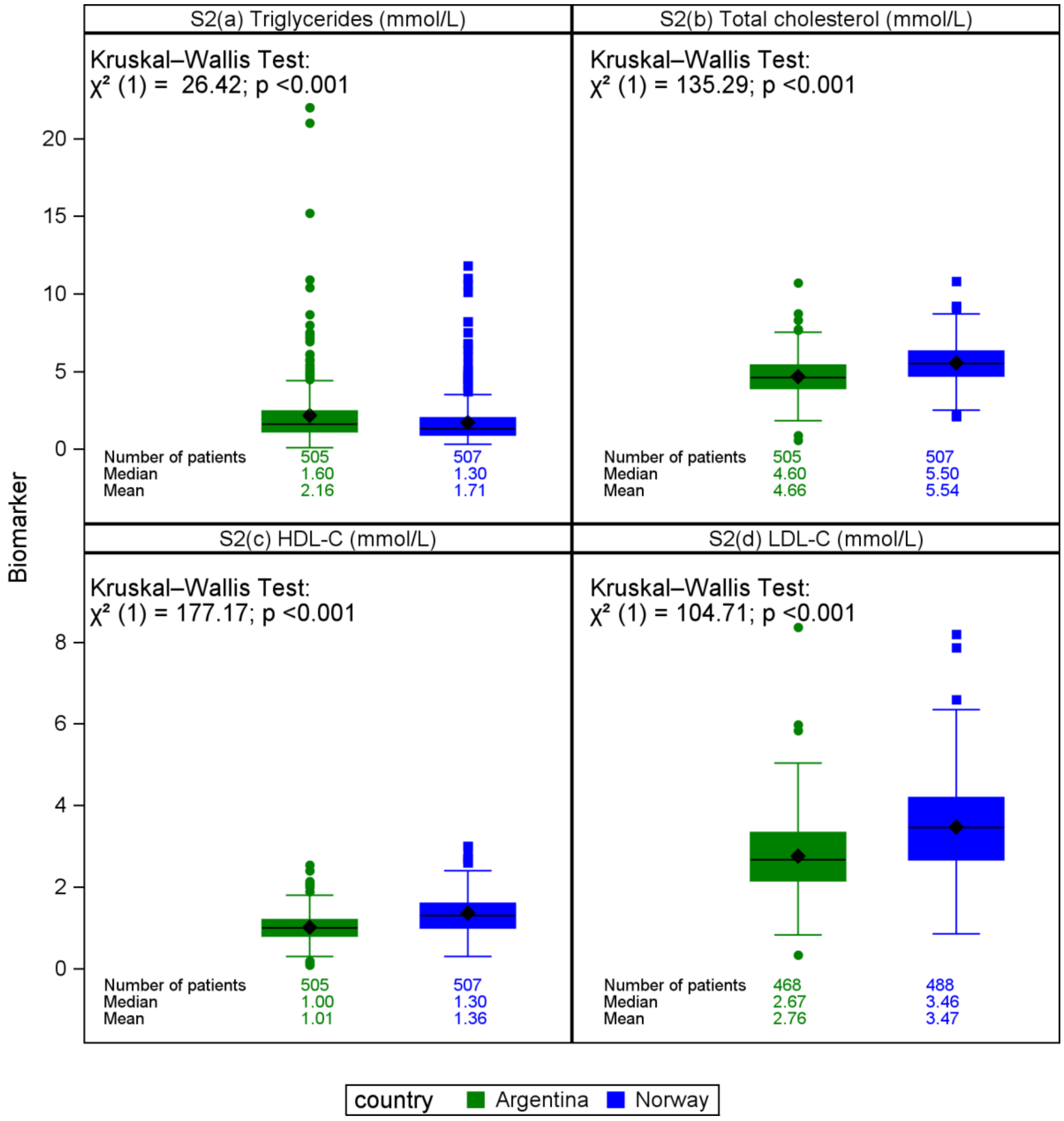

<u>Figure S3 a-b-c.</u> Scatter plots demonstrating correlations between log_e transformed values of angiopoietin-like 4 (ANGPTL4) and log_e transformed values of B-type natriuretic peptide (BNP) in a) the Argentinians, b) the Norwegians, and c) Argentinians and Norwegians combined, matched by propensity scoring according to age, gender and statin use. Solid line is fitted regression line; dotted lines are the 95% prediction interval.

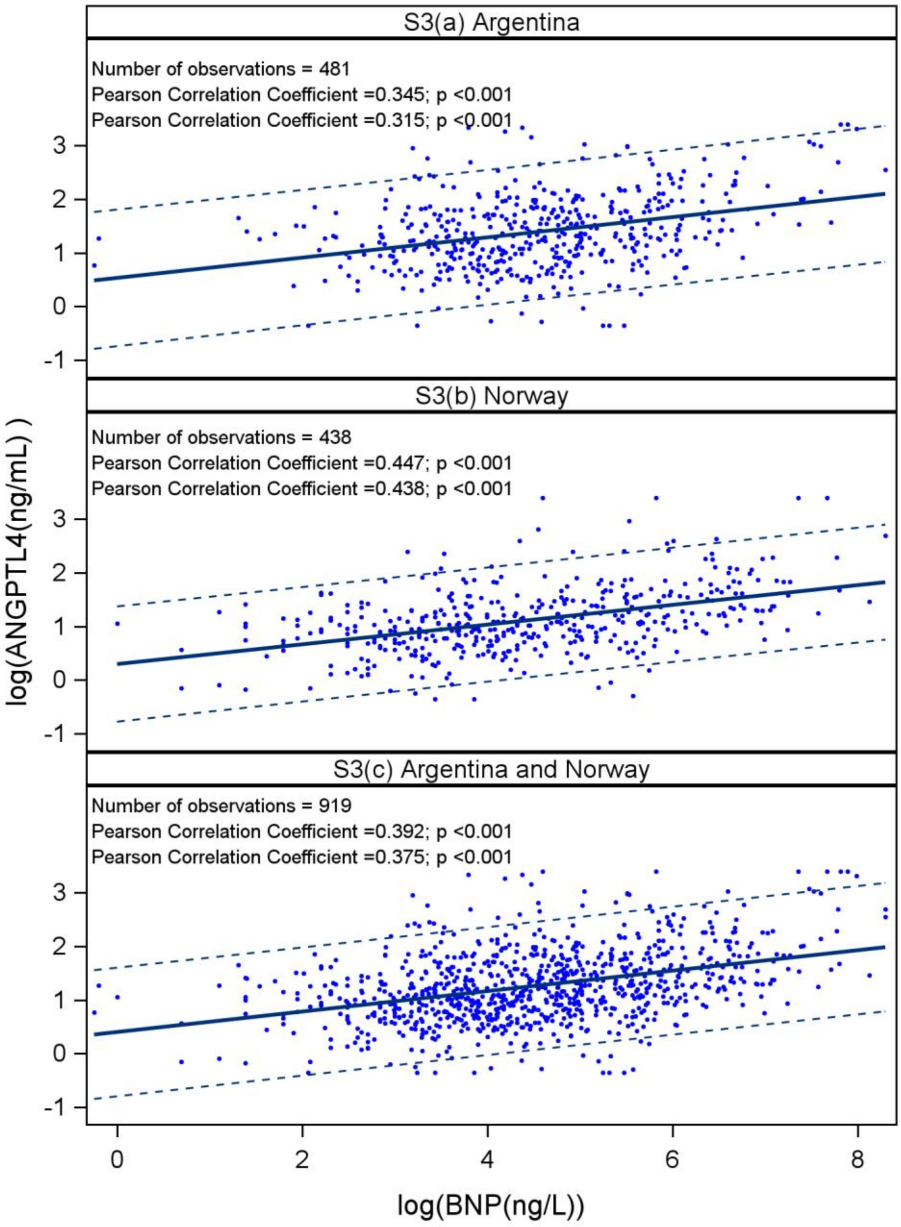

Figure S4 a-b-c. Scatter plots demonstrating correlations between $\log_e$ transformed values of angiopoietin-like 4 (ANGPTL4) and $\log_e$ transformed values of troponin-T (TnT) in a) the Argentinians, b) the Norwegians, and c) Argentinians and Norwegians combined, matched by propensity scoring according to age, gender and statin use. Solid line is fitted regression line; dotted lines are the 95% prediction interval.

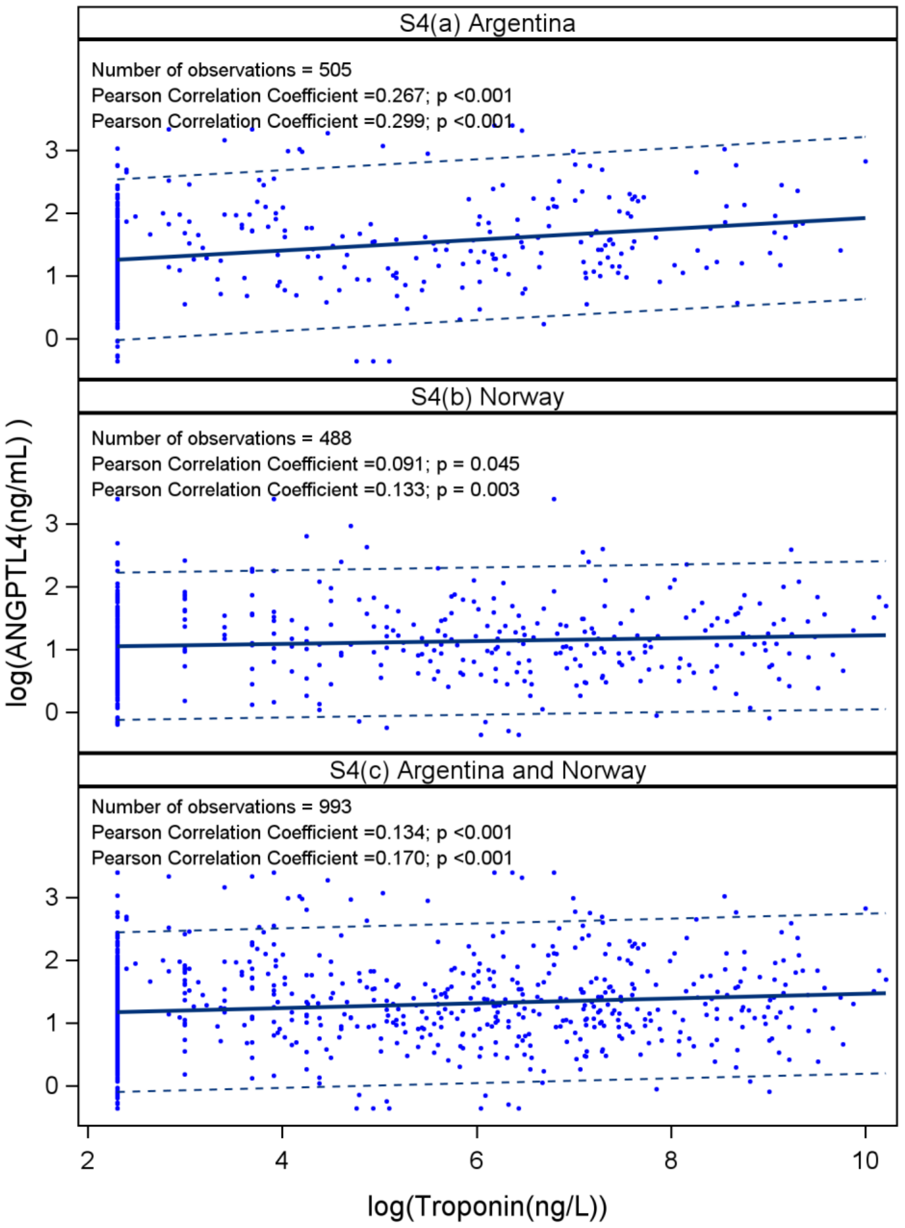

Figure S5. Kaplan-Meier plots describing the prognostic utility of angiopoietin-like 4 (ANGPTL4) for 2 years' all-cause mortality, arranged according to its quartiles in a) 504 Argentinian and b) 488 Norwegian patients admitted with acute chest pain of suspected coronary origin and matched by propensity scoring according to age, gender and statin use.

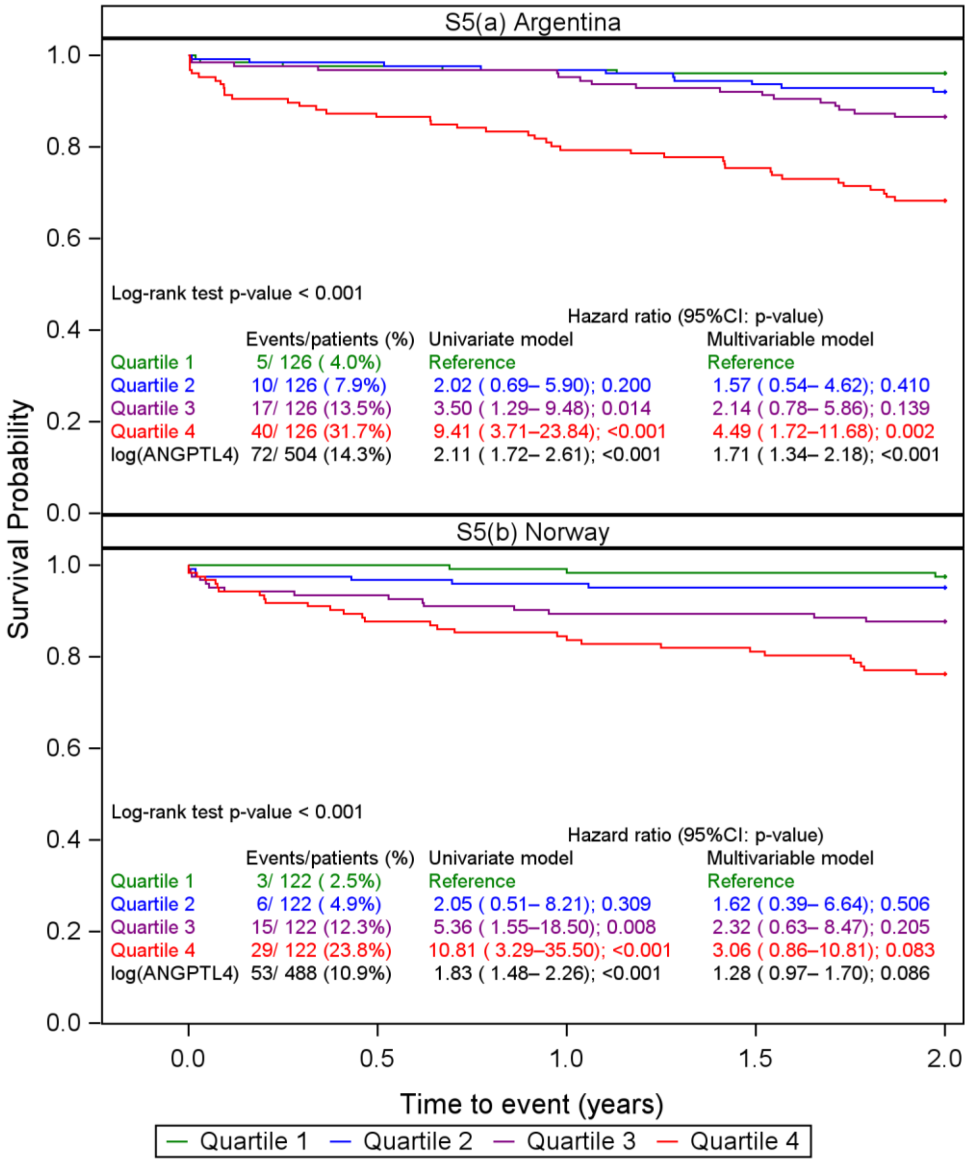

Figure S6. Kaplan-Meier plots describing the prognostic utility of B-type natriuretic peptide (BNP) for 2 years' all-cause mortality, arranged according to its quartiles in a) 505 Argentinian and b) 476 Norwegian patients admitted with acute chest pain of suspected coronary origin and matched by propensity scoring according to age, gender and statin use.

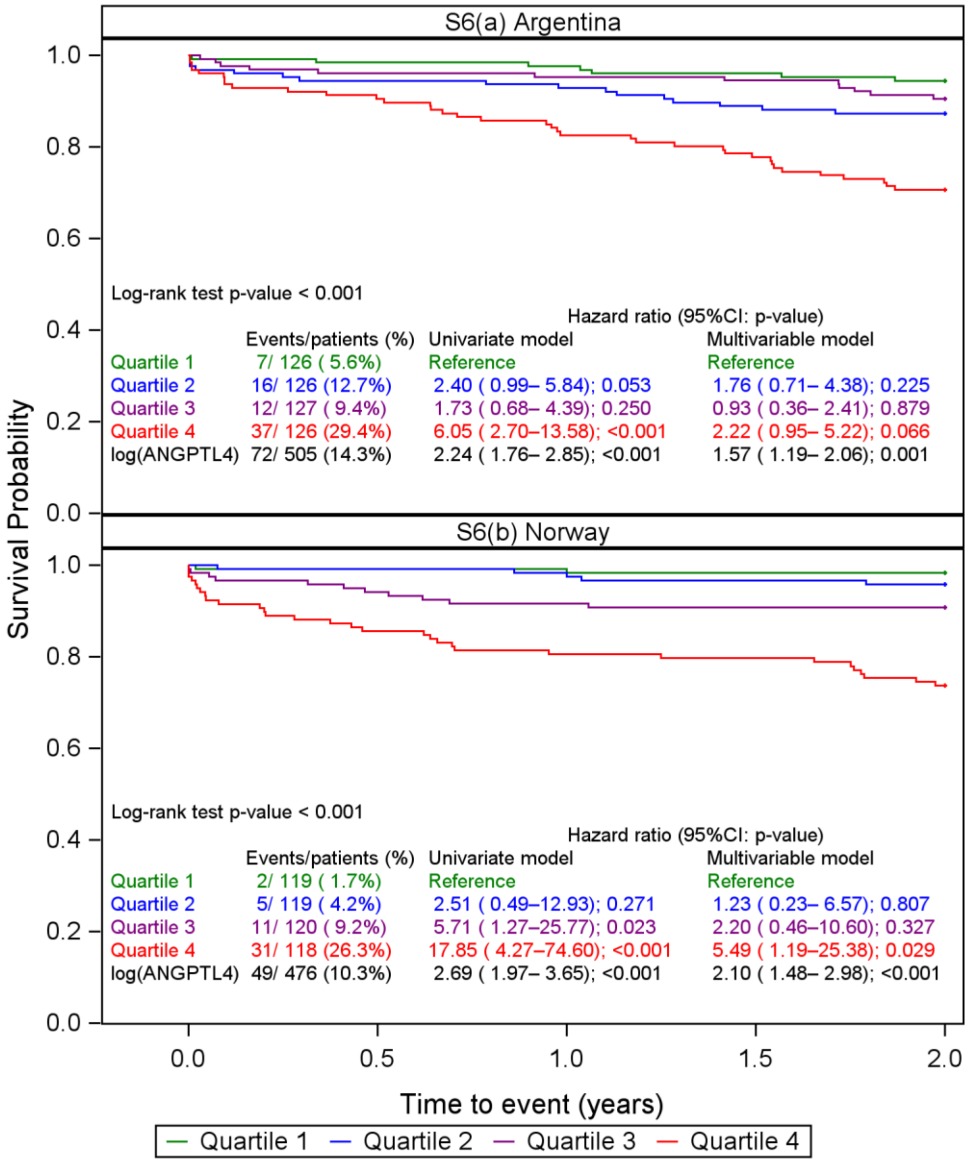

Figure S7. Kaplan-Meier plots describing the prognostic utility of angiopoietin-like 4 (ANGPTL4) for 5 years' cardiac mortality in the a) Argentinian population and for the composite endpoint of all-cause mortality or MI or stroke in the b) Norwegian population, arranged according to its quartiles in a) 504 Argentinian and b) 488 Norwegian patients admitted with acute chest pain of suspected coronary origin and matched by propensity scoring according to age, gender and statin use.

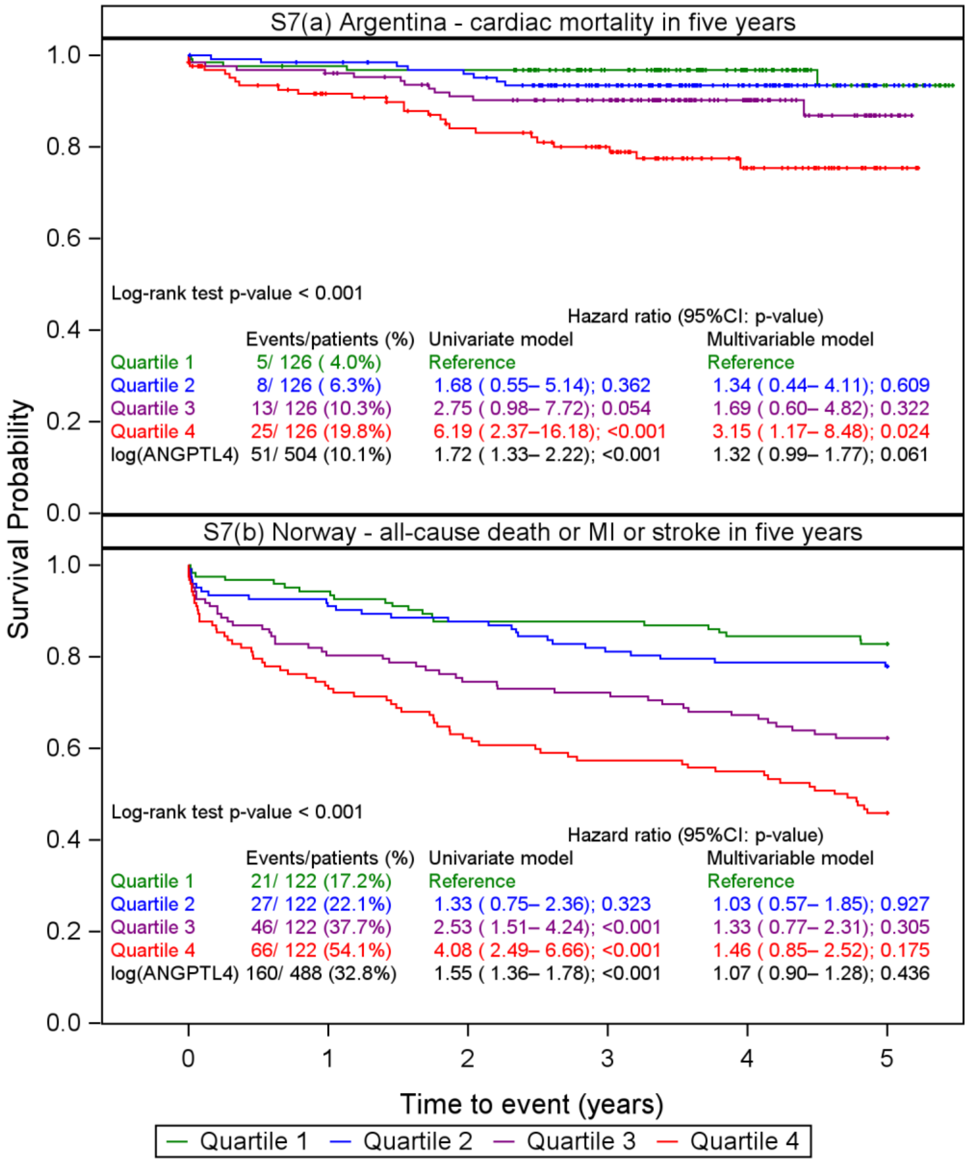

Figure S8. Kaplan-Meier plots describing the prognostic utility of brain natriuretic peptide (BNP) for 5 years' cardiac mortality in the a) Argentinian population and for the composite endpoint of all-cause mortality or MI or stroke in the b) Norwegian population, arranged according to its quartiles in a) 504 Argentinian and b) 488 Norwegian patients admitted with acute chest pain of suspected coronary origin and matched by propensity scoring according to age, gender and statin use.

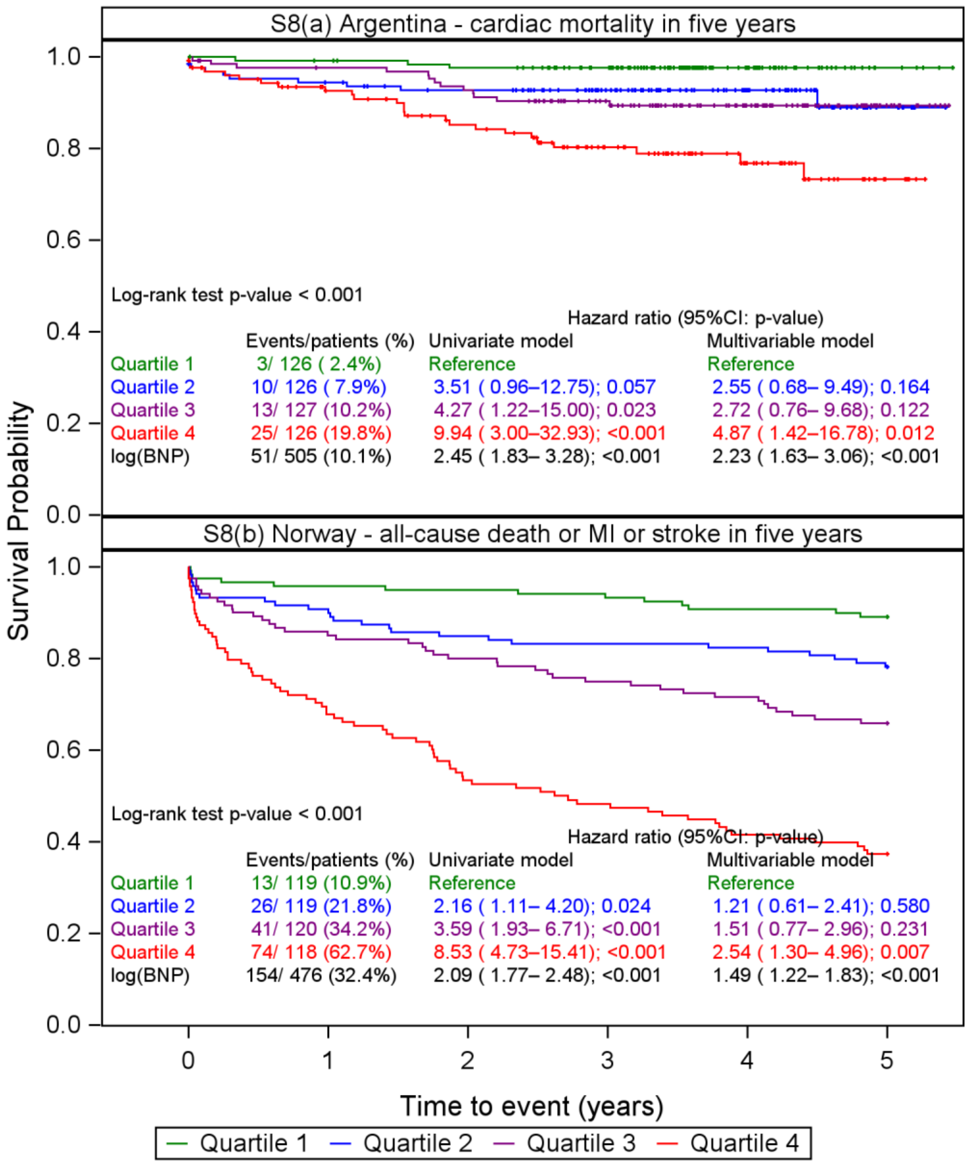

Figure S9 a-b-c. Scatter plots demonstrating correlations between $\log_e$ transformed values of angiopoietin-like 4 (ANGPTL4) and triglycerides (TG) in a) the Argentinians, b) the Norwegians, and c) Argentinians and Norwegians combined, matched by propensity scoring according to age, gender and statin use. Solid line is fitted regression line; dotted lines are the 95% prediction interval.

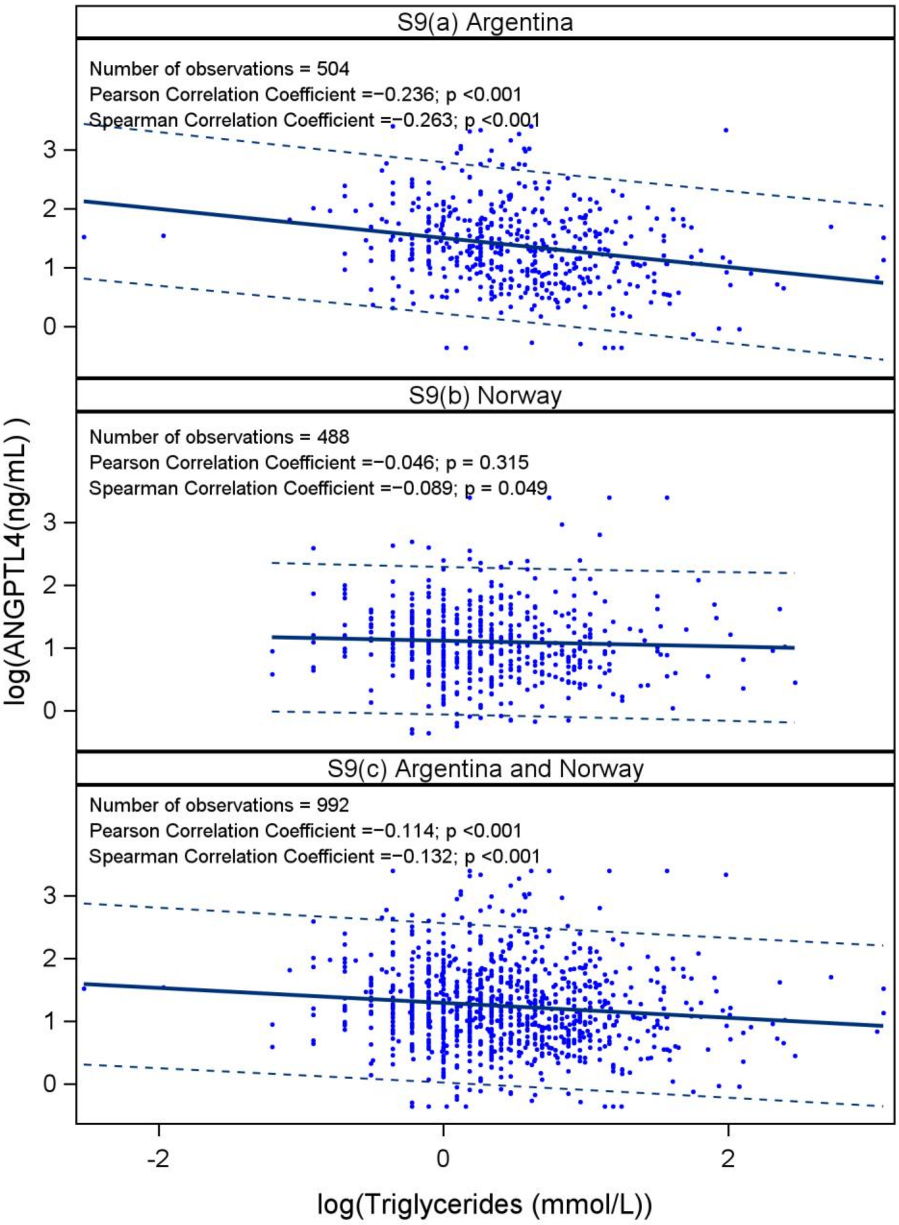

Figure S10 a-b-c. Scatter plots demonstrating correlations between $\log_e$ transformed angiopoietin-like 4 (ANGPTL4) and total cholesterol (TC) in a) the Argentinians, b) the Norwegians, and c) Argentinians and Norwegians combined, matched by propensity scoring according to age, gender and statin use. Solid line is fitted regression line; dotted lines are the 95% prediction interval.

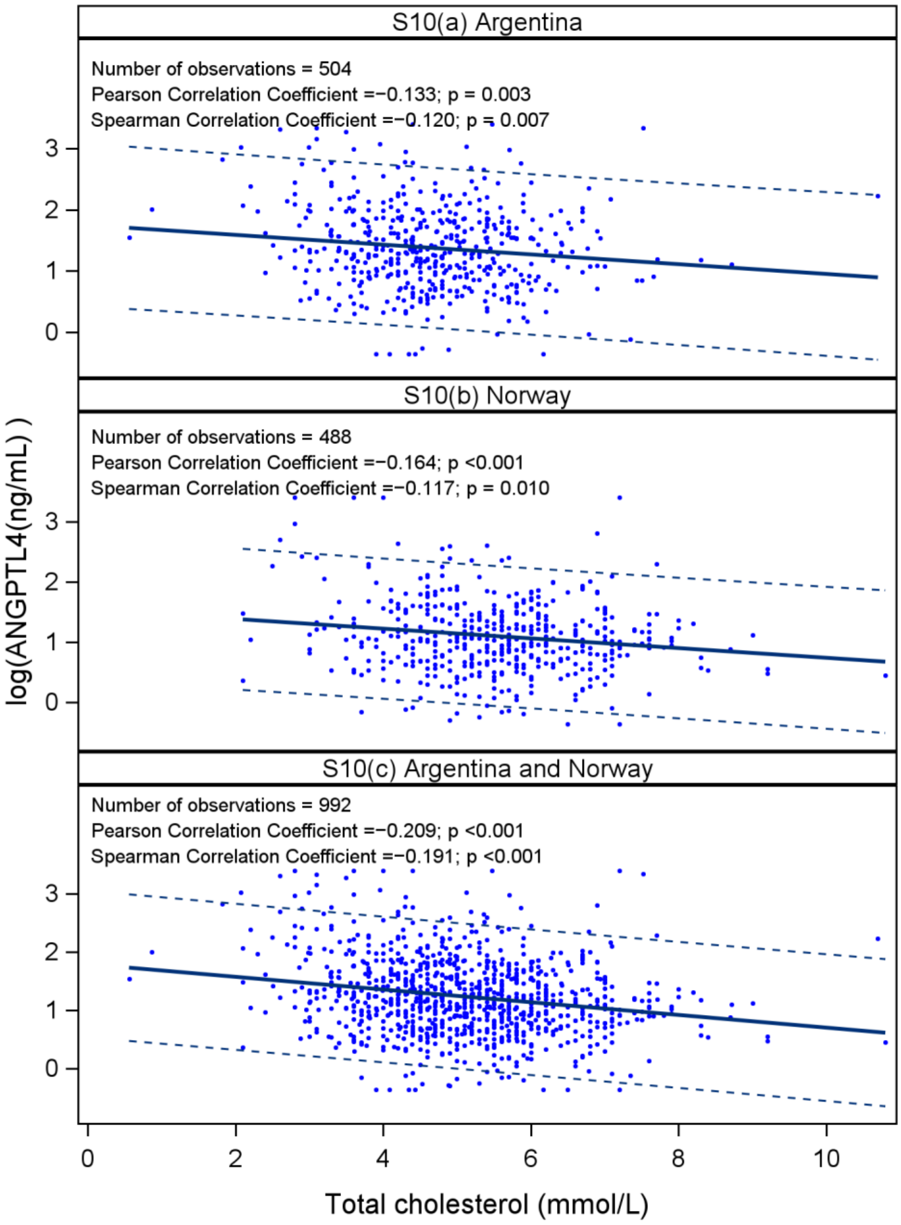

Figure S11 a-b-c. Scatter plots demonstrating correlations between $\log_e$ transformed angiopoietin-like 4 (ANGPTL4) and low-density lipoprotein cholesterol (LDL-C) in a) the Argentinians, b) the Norwegians, and c) Argentinians and Norwegians combined, matched by propensity scoring according to age, gender and statin use. Solid line is fitted regression line; dotted lines are the 95% prediction interval.

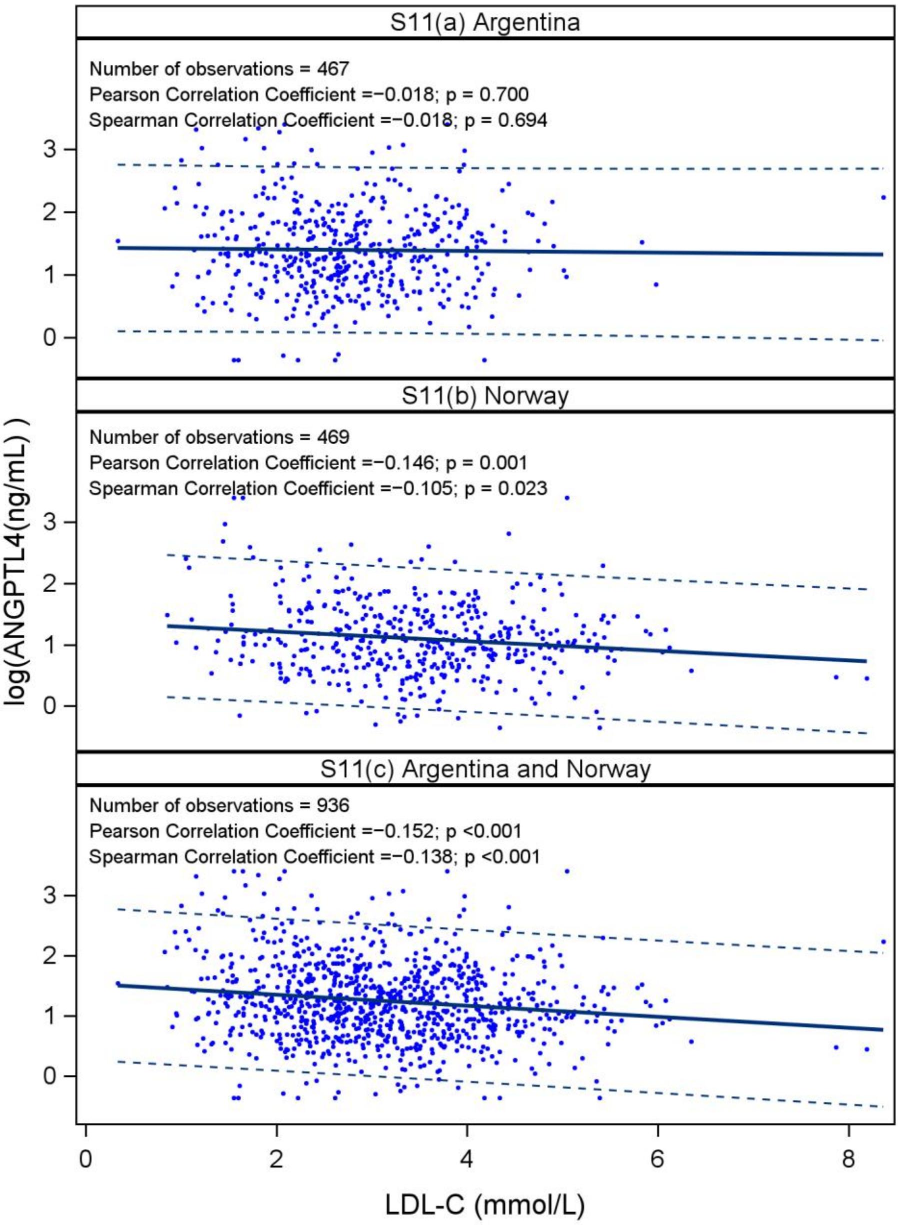

Figure S 12 a-b-c. Scatter plots demonstrating correlations between $\log_e$ transformed angiopoietin-like 4 (ANGPTL4) and high density lipoprotein cholesterol (HDL-C) in a) the Argentinians, b) the Norwegians, and c) Argentinians and Norwegians combined, matched by propensity scoring according to age, gender and statin use. Solid line is fitted regression line; dotted lines are the 95% prediction interval.

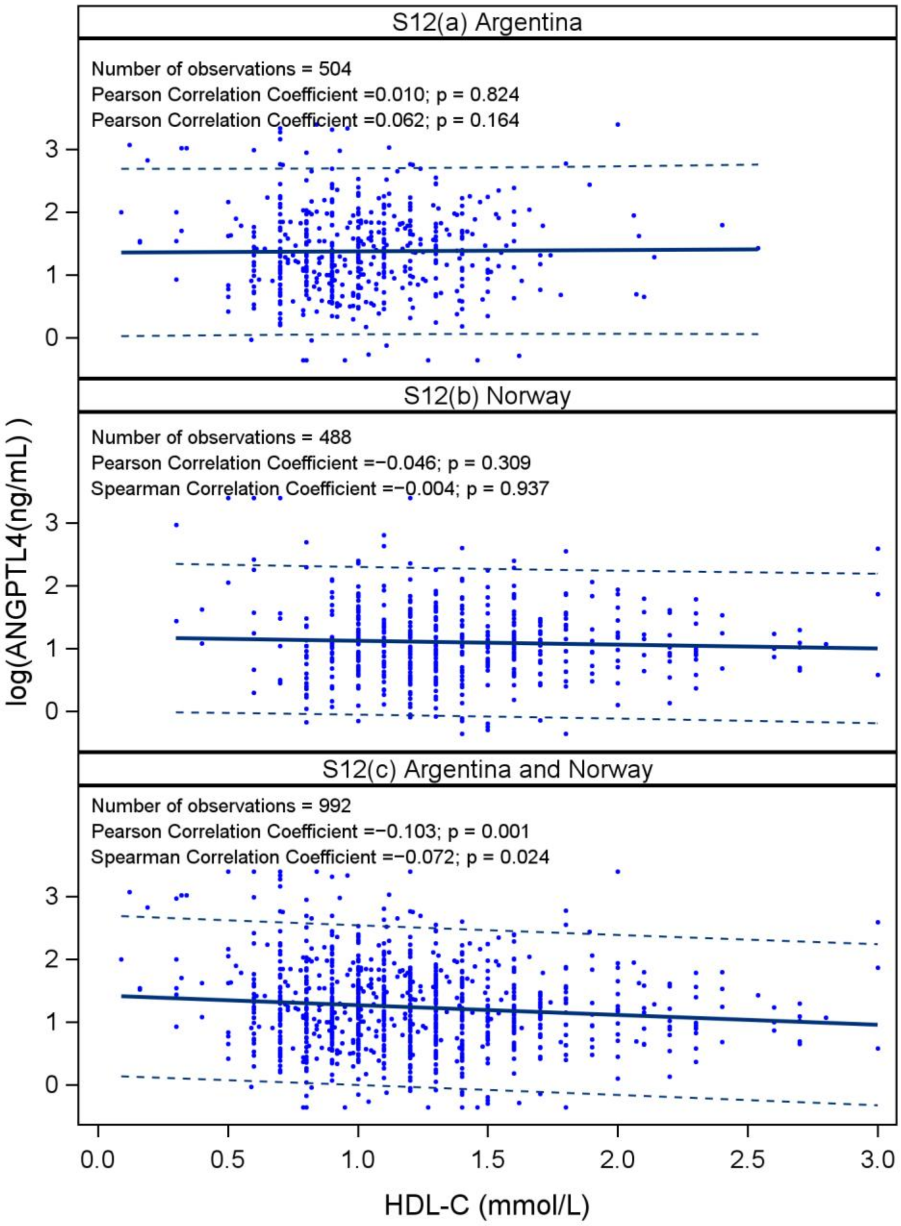

Supplement: Supplementary file 1 [file ijms-27-09966-s001.zip › ijms-4583659-supplementary.pdf]
